# Stability of Proteins in Dried Blood Spot Biobanks*[Fn FN2]

**DOI:** 10.1074/mcp.RA117.000015

**Published:** 2017-05-13

**Authors:** Johan Björkesten, Stefan Enroth, Qiujin Shen, Lotta Wik, David M. Hougaard, Arieh S. Cohen, Lene Sörensen, Vilmantas Giedraitis, Martin Ingelsson, Anders Larsson, Masood Kamali-Moghaddam, Ulf Landegren

**Affiliations:** From the ‡Department of Immunology, Genetics and Pathology, Science for Life Laboratory, Uppsala University, Uppsala, Sweden;; §Danish Center for Neonatal Screening, Statens Serum Institut, Copenhagen, Denmark;; ¶Centre for Inherited Metabolic Diseases, Karolinska University Hospital, Stockholm, Sweden;; ‖Department of Public Health and Caring Sciences, Uppsala University, Uppsala, Sweden;; **Department of Medical Sciences, Uppsala University, Uppsala, Sweden

## Abstract

An important motivation for the construction of biobanks is to discover biomarkers that identify diseases at early, potentially curable stages. This will require biobanks from large numbers of individuals, preferably sampled repeatedly, where the samples are collected and stored under conditions that preserve potential biomarkers. Dried blood samples are attractive for biobanking because of the ease and low cost of collection and storage. Here we have investigated their suitability for protein measurements. Ninety-two proteins with relevance for oncology were analyzed using multiplex proximity extension assays (PEA) in dried blood spots collected on paper and stored for up to 30 years at either +4 °C or −24 °C.

Our main findings were that (1) the act of drying only slightly influenced detection of blood proteins (average correlation of 0.970), and in a reproducible manner (correlation of 0.999), (2) detection of some proteins was not significantly affected by storage over the full range of three decades (34 and 76% of the analyzed proteins at +4 °C and −24 °C, respectively), whereas levels of others decreased slowly during storage with half-lives in the range of 10 to 50 years, and (3) detectability of proteins was less affected in dried samples stored at −24 °C compared with at +4 °C, as the median protein abundance had decreased to 80 and 93% of starting levels after 10 years of storage at +4 °C or −24 °C, respectively. The results of our study are encouraging as they suggest an inexpensive means to collect large numbers of blood samples, even by the donors themselves, and to transport, and store biobanked samples as spots of whole blood dried on paper. Combined with emerging means to measure hundreds or thousands of protein, such biobanks could prove of great medical value by greatly enhancing discovery as well as routine analysis of blood biomarkers.

It may be said that every individual is a medical experiment that potentially could help define biomarkers to diagnose diseases by combining analysis of biological samples with information about medical conditions and environmental factors. These analyses will require samples from very large numbers of individuals, but it is also important to secure multiple samples from each individual. The availability of consecutive samples increases the chance that samples will be available from a time point optimal for diagnosis, before a disease becomes clinically manifest, and it also allows trends to be monitored over time where each individual serves as his or her own control.

Blood is an ideal matrix for molecular analysis because of its accessibility and the fact that disease processes anywhere in the body are potentially reflected in altered levels of molecules released into circulation. Traditional blood sampling by venipuncture and centrifugation to obtain plasma requires trained personnel and laboratory equipment, and the samples are typically stored as tubes frozen at −80 °C, taking up considerable space in energy-consuming freezers.

Dried blood spots (DBS),^1^ offer several advantages over conventional blood sampling. These advantages include convenient collection during routine blood sampling or even via a finger prick by a lancet, permitting home sampling, and DBS can be sent to a lab or a biobank by regular mail, and be stored without taking up much space (1–4). These features can significantly reduce the cost of blood sampling and storage, and increase convenience for sample donors. A major limitation of DBS is the small amount of sample material collected in this way. Nonetheless, it has been demonstrated that protein levels can be measured from DBS samples (5–7). In particular, 92-plex protein abundance can be measured from a 1.2-millimeter diameter punch from a DBS using the proximity extension assay (PEA) technique (3, 8). This enables measurement of several hundred proteins from a single DBS. Similar levels of multiplexing have been achieved with state of the art mass spectrometry by using an entire DBS (6, 7). An earlier PEA-based investigation revealed relative increases of some proteins in DBS compared with conventional centrifuged plasma (8)—a likely consequence of the contribution of proteins from blood cells in the DBS. Normal levels of leukocytes range between 4000–11,000 cells/μl (9), and the wide range may contribute to variability of protein levels in DBS samples compared with the more conventional plasma samples.

A study of the stability of RNA in DBS revealed no significant reduction of levels of β-actin or total RNA during 20 years of storage at +4 °C (10). To the best of our knowledge no corresponding information is available regarding the stability of proteins in DBS. The amounts of a majority of 169 investigated peptides were preserved during 154 days of storage at −20 °C to 37 °C when analyzed by mass spectrometry, demonstrating stabilities largely independent of storage temperature over this limited period (6). Here we have investigated both immediate effects on immunodetection of proteins using PEA by the drying process, as well as effects of long-term storage. Using a multiplex analysis of 92 proteins related to oncology, we compared results for wet *versus* dried samples of whole blood, blood plasma, or a dilution series of the 92 target proteins in purified form. We furthermore investigated DBS from newborn screening programs stored for up to 30 years either at +4 °C or at −24 °C, and we compared protein measurements for liquid plasma samples stored at −70 °C for similar lengths of time, obtained from adult men. Our results demonstrate that drying only slightly but consistently influenced detectability, decay of protein detectability over time varied among the protein analyzed, and lowering the storage temperature reduced the rate of decay of proteins stored as DBS.

## MATERIALS AND METHODS

### 

#### 

##### Samples for Evaluating the Drying Process

One EDTA blood sample was obtained from a volunteer blood donor at Uppsala University Hospital. DBS samples were prepared by pipetting ∼25 μl EDTA blood onto a Whatman DMPK-C sample collection card (GE Healthcare). The drops of blood were allowed to hang from the tip of the pipette and touch the paper to be absorbed, taking care not to touch the paper with the pipette tip. The DBS samples were allowed to dry for 3h at room temperature (RT). A few milliliters of the original EDTA blood were meanwhile kept at +4 °C. The rest of the EDTA blood sample were centrifuged for 15 min at 2000 × *g*, and plasma was decanted and collected. Dried plasma spots (DPS) were prepared in the same way as the DBS. 1.2 mm in diameter disks were punched out from the DBS and DPS with a Uni-Core^TM^ 1.2 mm micro puncher (GE Healthcare). Punching from an empty area of the sampling card was performed in between every sample to limit carry-over contamination between samples. The abundance of 92 proteins related to oncology was measured from 1.2 mm disks, punched from DBS and DPS and in 1 μl aliquots of wet blood and wet plasma (see Protein Abundance Measurements below for details).

A pool of 92 recombinant antigens targeted by the assays included in the Proseek Multiplex Oncology II panel (supplemental Table S1) was received from Olink Proteomics AB in Uppsala, Sweden. The concentration of each protein in the stock solution was 1 μg/ml. A series of 10-fold dilutions were made in PBS supplemented with 0.1% bovine serum albumin, and dried samples were prepared by applying 2.5 μl of proteins at concentrations ranging from 500 ng/ml to 5 pg/ml to Whatman 903 Protein Saver cards (GE Healthcare). The area of the resulting spots was ∼12.5 mm^2^ (calculated based on the average diameters of the spots). The protein spots were dried at RT for ∼3 h before three 1.2 mm in diameter disks were punched out from the middle of the spots for analysis. The estimated amount of plasma present in one punch was ∼0.2 μl (calculated from the applied volume, the measured size of the spot, and the assumption that the concentration of proteins in the spot is essentially homogenous across the DBS). For wet samples the analyzed volume was 1 μl. For the dilution series of the pool of pure proteins, five times lower concentrations of wet samples ranging from 100 ng/ml to 1 pg/ml were used to compensate for this difference. Also 1.2 mm diameter paper punches with no sample material were analyzed to evaluate potential influence on protein measurement by the paper material alone.

##### Samples from the Swedish and Danish Newborn Screening Programs of Metabolic Disorders

DBS samples stored for 0.5, 10.5, 20.5, 30.5, and 40.5 years from five newborn individuals each were collected from the Swedish newborn screening program for inherited metabolic disorders at the Karolinska University Hospital in Solna, Sweden. Also, fresh DBS controls were collected from five individuals and analyzed within a few weeks from sampling (stored at +4 °C before analysis). All DBS were collected in the month of July in 1975 to 2015 except for the fresh controls that were collected in December 2015. The paper cards used for sampling have not been the same from 1975 until today. No documentation was found of the type of paper used between 1975 and 1981. From 1981 until 2001 Schleicher & Schuell number 2992 was used. Between 2001 and 2009 Schleicher & Schuell number 903 was used and since 2009 until today Ahlströmer 226 is used. All paper types are expected to have very similar properties. Also, the storage conditions of the biobank have changed over time. Between 1975 and 1980 the Swedish biobank was kept at RT. From August 1981, the biobank was relocated to a cold room with a temperature of +4 ± 2 °C and humidity less than 70%. During 1994 the samples were relocated to a new cold room with the same temperature. From 1994 to 1996 the humidity of the cold room was uncontrolled but in 1996 dehumidification equipment was installed to maintain the humidity below 30% and since then the biobank has been kept under these conditions. The DBS cards are stored directly on top of each other in bundles in plastic Ziploc bags.

DBS samples stored for 0.5, 10.5, 20.5, and 30.5 years from five individuals each were collected from the Danish newborn screening program for inherited metabolic disorders at Statens Serum Institut in Copenhagen, Denmark. Also, fresh DBS controls from five individuals were analyzed within a few weeks from sampling (stored at −20 °C before analysis). All Danish DBS were collected in the month of July in 1985 to 2015, except for the fresh controls that were collected in January of 2016. From 1985 until 1990 Schleicher & Schuell number 2992 was used, followed by Schleicher & Schuell number 903 between 1990 and 2009. From January 2010 until today Ahlströmer 226 has been used. The Danish biobank has been kept at −24 °C all along.

The samples received from both the Swedish and Danish biobanks were completely anonymized to us, we do not even know the gender of the newborns sampled.

##### Samples from the Uppsala Longitudinal Study of Adult Men (ULSAM) Biobank

Plasma and/or serum samples from six groups stored for ∼45, 22, 16, 13, 7, and 2 years from five individuals with ages of 50, 71, 77, 82, 86, and 91 years, respectively, were collected from the ULSAM biobank in Uppsala, Sweden (supplemental Table S2). The individuals sampled were not the same across the time series. The liquid samples had been stored at −70 °C. It is not known whether some of the samples had been previously thawed. Paired fresh serum and plasma samples from five men from each of the 50, 60, 70, 80, and 90 (±5 years) years age groups were received from the Department of Clinical Chemistry at the Uppsala University Hospital, Sweden. These fresh control samples were used to normalize for the age of individuals and sample type (plasma or serum). They were also used as a standard to estimate whether and if so by how much the protein levels had changed in the samples stored for different lengths of times at −70 °C.

##### Evaluation of Protein Homogeneity Across DBS

The 1.2 mm diameter punched out DBS disks used in this study were taken from the centers of the DBS. To evaluate the homogeneity of protein distributions across DBS, 1.2 mm punches were collected from the middle and edges from two equivalent DBS, prepared from a single finger prick from one donor. In total six punched out disks were collected and analyzed from these two DBS (supplemental Fig. S1*A*).

##### Protein Abundance Measurements

Using PEA technology we measured the abundance of sets of proteins in DBS, plasma, serum, and in recombinant antigen samples. PEA has been developed and commercialized as 92-plex Proseek Multiplex protein panels by Olink Proteomics AB, Uppsala, Sweden (8). PEA utilizes pairs of affinity probes to measure levels of the targeted proteins. The affinity probes consist of antibodies with conjugated oligonucleotides having complementary 3′ ends. Upon target recognition by pairs of such probes the conjugated oligonucleotides can be extended by a polymerase. The amounts of extended oligonucleotides for each antibody pair is used as a measure of numbers of detected proteins, as recorded by real-time PCR using the 96.96 Dynamic Array^TM^ IFC microfluidic chip based qPCR system (Biomark HD, Fluidigm). The data was normalized using an internal extension control to adjust for technical intra-run variation and shifted using a correction factor to account for normal background noise. The final values are called Normalized Protein eXpression (NPX) values, an arbitrary value on a log 2-scale. High NPX values correspond to high protein abundance. Three negative control samples were used to define the limit of detection (LOD) as protein measurement exceeding the average background by three times its standard deviation. This was performed for each protein in each run. Validation data for all proteins, including lower and upper limit of quantification, can be found at the manufacturer's webpage (www.olink.com).

To evaluate the effects of drying on protein detectability using blood and plasma samples, we selected the Proseek Multiplex Oncology I v2 panel. For the analysis of a dilution series of recombinant antigens, biobank samples, samples for evaluation of homogeneity across DBS, and fresh controls, the Proseek Multiplex Oncology II panel was used. Both panels measure 92 proteins of relevance for oncology and they include four control assays (supplemental Table S1 and S3).

Proseek Multiplex panels are primarily developed and validated to measure proteins in 1 μl aliquots of plasma. However, the assays have been previously shown to allow convenient detection of proteins contained in 1.2 mm diameter DBS disks with only minor changes to the general protocol (8). Instead of adding 1 μl plasma to the 96-well PCR plate, a punched-out DBS disk is added together with 1 μl negative control solution (Olink Proteomics). Thereafter the protocol is the same as for liquid samples but attention is taken not to disturb the paper in the bottom of the well after the pre-extension step when sample is transferred to the microfluidic chip. If fibers from the paper are transferred together with the sample the microfluidic chip used for real-time PCR readout might get clogged.

When analyzing the DBS samples stored at −24 °C five samples failed to pass the Proseek Multiplex quality control. These five values were spread out over the different storage times analyzed. When examining the values for these samples it was obvious that the levels from these samples stood out from the others. The five samples were reanalyzed successfully and the new values were used for further analysis.

##### Statistical Analysis

All statistical analyses were performed in R (R Development Core Team, 2014). Description on how to calculate NPX values can be found at the Olink website (www.olink.com). LOD values (log_2_ scale) were subtracted from NPX values (log_2_ scale) to achieve NPX signal to noise (S/N) values. Normalization for the paper material included when analyzing DBS were carried out by first subtracting LOD values (log_2_ scale) from the background paper material NPX values (log_2_ scale), and second subtract the resulting values from the LOD normalized DBS NPX values (log_2_ scale). Negative S/N values were set to zero for visualizations and calculations. Calculated correlations between replicates of wet or dried samples of EDTA blood or EDTA plasma for all 92 measured proteins were estimated with the nonparametric rank based Spearman method. Linearized protein abundances (2^NPX^) were used to calculate average fold changes from duplicate measurements of individual protein levels in blood compared with in plasma. To visualize the distributions of NPX S/N values for DBS samples stored for different times under different conditions an R package called Beeswarm was used. Differences between these groups of samples with 460 data points each were evaluated with independent 2-group t-tests. Effects of storage time on individual proteins were examined by fitting linear regression models with years of storage as variable and the protein abundance levels (NPX values) as responses. The significances of these models were evaluated by an analysis of variance (ANOVA) tests. Half-lives were calculated for the 20 proteins that were significantly decreased after testing for multiple hypotheses (Bonferroni, *p* value < 0.05/92) in samples stored at +4 °C and −24 °C. The half-lives were estimated from the slopes of the linear models as the time to decrease by one NPX unit (log_2_ scale). To achieve values of relative signals for individual proteins the NPX values were first transformed to linear scale (2^NPX^). Measured protein abundances in samples stored for 10, 20, and 30 years were then divided by the corresponding protein abundances in samples stored for 0.5 years to calculate relative abundances upon storage. Significances of differences between relative abundances of groups of 92 proteins were calculated with independent 2-group *t*-tests.

To evaluate the homogeneity of protein distribution across DBS samples, punches were collected from the edges (*n* = 4) or middle (*n* = 2) of two DBS obtained from the same individual (supplemental Fig. S1*A*). Independent 2-group t-tests were performed individually for all 92 proteins measured. Correlations between different replicates were calculated by the nonparametric rank based Spearman method.

To separate the effects of ages of the sampled individuals from effects of storage-time in the ULSAM-cohort, protein abundances were first adjusted by linear regression, taking into account both the individual age and the sample source (plasma or serum) in models that were built from fresh-frozen samples. The significances of these models were evaluated by ANOVA tests. The levels of all proteins (regardless of storage-time) with nominally significant (*p* < 0.05) contributions were subsequently adjusted for age and/or plasma/serum by removing the contribution using the beta (β)-coefficient(s) from the linear models. Finally, contribution of storage-time in the ULSAM-cohort was estimated using linear regression and ANOVA with Bonferroni adjusted *p* values as above but with the adjusted protein abundance levels as input.

##### Ethical Considerations

The study was conducted in accordance with the Declaration of Helsinki. For the fresh adult samples, the study was approved by the Regional Ethics Committee at Uppsala University for control samples (Dnr. 01–367), and for longitudinal ULSAM samples (Dnrs.: 251/90, 97–329, 02–605, 2007/338 and 2013/350). All ULSAM samples were anonymized for the current study. The Danish and Swedish neonatal biobank DSB samples were received fully anonymized including the gender of the subjects, in accordance with the Danish “Act on Research Ethics Review of Health Research Projects,” and Swedish law (2003:460) for Ethical Review of Research Involving Humans, respectively.

## RESULTS

### 

#### 

##### Effect on Protein Detectability by Drying

**Fig. 1.**
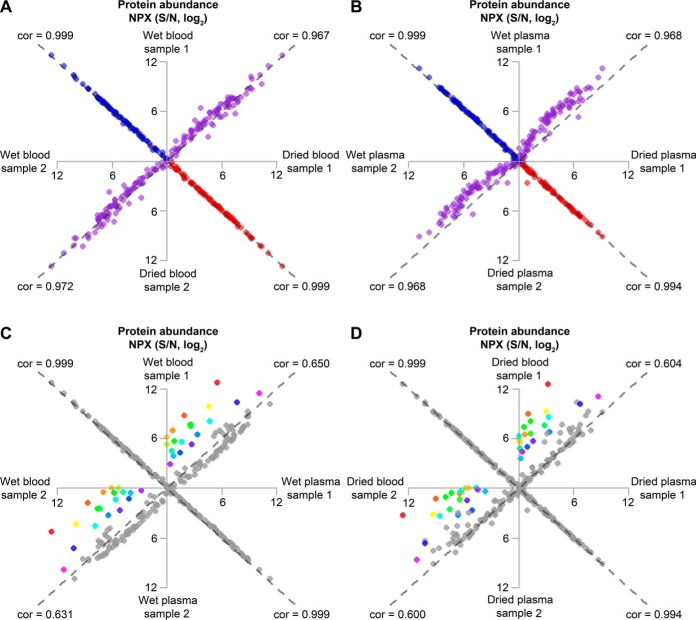
**Abundances of 92 proteins of relevance in oncology, measured with PEA technology from whole blood or plasma samples in either dried or liquid form.** Duplicate samples were used to evaluate relations of protein levels in wet and in dried samples, and in blood and plasma samples. The results are visualized in scatterplots, containing four correlations each. *A*, Correlations of protein abundance between wet and dried EDTA blood samples (purple) and technical replicates for wet EDTA blood (blue) and dried EDTA blood (red) samples. *B*, Correlations of protein abundance between wet and dried EDTA plasma (purple) and technical replicates for wet EDTA plasma (blue) and dried EDTA plasma (red) samples. *C*, Correlations between protein levels in wet EDTA blood and wet EDTA plasma (colored/gray) and technical replicates for wet EDTA blood and wet EDTA plasma (gray). *D*, Correlations between dried EDTA blood and dried EDTA plasma (colored/gray) and technical replicates for dried EDTA blood and dried EDTA plasma. The 20 proteins exhibiting the highest average fold differences between whole blood and plasma (farthest away from the dashed gray line of equivalence) in the comparisons between protein measurements in wet blood and plasma in (*C*) were assigned individual colors, and the same colors were applied for the dried blood and plasma samples in (*D*) and in supplemental Table S4. The results demonstrate that this group of colored proteins exhibited similar patterns of elevated levels in blood compared with in plasma in both wet and dried samples. All other protein measurements in the comparisons between blood and plasma are colored gray. The calculated correlation values are based on the rank based Spearman method. The gray dashed lines correspond to the equivalence between compared samples (*x* = *y*). Individual levels of LOD (log_2_-scale) have been subtracted from NPX values (log_2_-scale) for each protein. The dried samples were also normalized for the shift in background levels measured from the paper material control sample. Any resulting negative values (signals below LOD) were set to zero.

Deposition of samples of blood or plasma to be dried on paper is an attractive means to collect and store patient samples, provided that the results of analysis are not compromised. The first aspect to be evaluated was the effect on protein detectability by the process of drying of samples on paper. Three different kinds of samples were investigated; Ethylenediaminetetraacetic acid (EDTA) blood, EDTA plasma and a dilution series prepared from a pool of 92 recombinant antigens. These were all analyzed both in the dried and wet states. No adjustments for input sample amounts were made for EDTA blood or EDTA plasma when comparing dried and wet samples. All samples were analyzed in duplicates. Correlations between replicates, between wet and dried samples, and between blood and plasma samples are all shown in four scatterplots, each reflecting four correlations, for all measurements of the 92 proteins with NPX values above the LOD (Fig. 1). The dried samples were normalized for a slight shift in background levels measured for paper samples. This normalization only marginally improved the correlations. The measured protein levels correlated very well between wet and dried samples (0.967 and 0.972; rank based Spearman method) but correlation coefficients were nonetheless lower than those of technical replicates for EDTA blood and EDTA plasma (0.994 and 0.999, respectively) (Fig. 1*A* and 1*B*). No significant difference were observed in the numbers of undetectable proteins in either dried or wet, blood or plasma samples. Only a small group of 3 or 4 proteins were undetectable in each sample, from a total of 7 different proteins that were undetectable in at least one of the samples.

Very similar patterns were demonstrated in wet and dried samples for both EDTA whole blood (Fig. 1*A*) and EDTA plasma (Fig. 1*B*), revealing only minor effects on protein detectability by the act of drying the samples. By contrast, as expected we observed clear differences between blood and plasma both in wet form (Fig. 1*C*) and as dry samples (Fig. 1*D*). The whole blood samples include red and white blood cell proteins, besides plasma proteins. The average fold change was calculated for each protein in the comparisons between wet blood and wet plasma. Fold change is interpreted as the perpendicular distance to the line of equivalence (dashed gray line) in the NPX plot, which is a log_2_-log_2_ plot of the measured protein levels. The 20 proteins exhibiting the greatest concentration ratio in wet blood *versus* plasma in Fig. 1*C* were assigned individual colors as shown in Fig. 1*C* and 1*D*. The identities of the color-coded proteins are presented in supplemental Table S4.

Only small differences were demonstrated when we evaluated wet and dried dilution series of pools of recombinant proteins, although for most proteins the difference between wet and dried samples exceeded the technical variation between the triplicate measurements (supplemental Fig. S2).

It is important to assess the homogeneity of protein distribution across a DBS if small portions of a DBS are to be used for analysis. We investigated the abundance of 92 proteins in punches taken either from the edges or the middle of two equivalent DBS (supplemental Fig. S1). The results demonstrate generally good correlation between results for protein measurements in samples from edges and middle parts of a DBS (supplemental Fig. S1*B*). Nonetheless, for eleven out of the 92 analyzed proteins minor, nominally significant concentration differences (*p* value < 0.05), were noted between samples taken from the edges and middle parts of the two DBS (supplemental Fig. S1*C*). For 10 of these proteins slightly higher levels were observed in samples collected from the edges rather than middle of the DBS. The differences between the groups were consistently very small, with average difference for the 10 proteins slightly more abundant in samples from the edges of the DBS ∼0.2 NPX or 15% higher than in those from the middle of the DBS. After correction for multiple hypothesis testing, none of the analyzed proteins differed significantly (Bonferroni, *p* value < 0.05/92). We conclude that the location of the punches in the DBS has negligible effects on protein levels.

##### Comparison of Long Term Storage Stability of Proteins Measured in DBS Stored at +4°C or −24°C

**Fig. 2.**
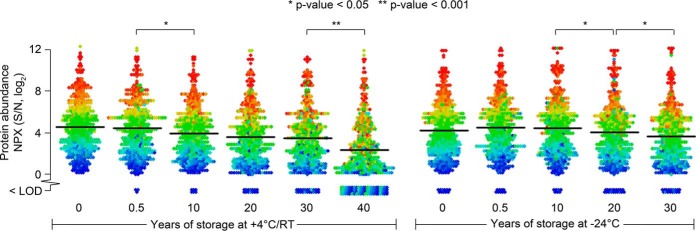
**Distribution of measurements for 92 proteins in DBS samples stored at +4 °C or −24 °C for up to 30 years.** Dried blood samples were grouped based on storage time and storage temperature, generating 11 groups, and for each group protein values are displayed for 5 individuals for a total of 460 data points in each group. For each protein the LOD in NPX values (log_2_-scale) has been subtracted from the measured NPX values (log_2_-scale). Any resulting negative values (undetectable levels) were set to <LOD. The distribution of data points in the different groups was visualized as bee swarm plots. Each of the 92 different proteins was assigned an individual color nuance on a scale from red to blue according to their average levels of expression in samples stored for 0 years. The assignment of colors was performed separately for samples stored at RT/+4 °C or at −24 °C. The line in each distribution represents the group average for all proteins (proteins below LOD were assigned a value of zero for this calculation). The samples stored for 40 years were first stored for 6 years at RT and subsequently for 34 years at +4 °C. All other samples were kept at +4 °C or −24 °C the entire storage time. Independent 2-group t-tests were used to evaluate the significance of differences between nearby groups. Significant differences (*p* value < 0.05) were demonstrated for four comparisons as indicated by asterisks. The most significant difference was observed between samples stored for 30 or 40 years at +4 °C and RT/+4 °C, respectively (*p* value = 10^−10^).

We investigated protein levels in DBS samples collected neonatally and stored for variable amounts of time at either +4 °C or −24 °C. Results were pooled for the 92 proteins measured in the five investigated samples for each time and temperature of storage, resulting in 460 data points in each storage group. Fig. 2 presents a bee swarm plot illustrating the distributions of NPX-values above LOD and the average values for the different groups. LOD-values were subtracted from NPX-values and any resulting negative values were reported as <LOD. For calculating group averages NPX-values <LOD were identified as zero. Independent 2-group t-tests were performed for groups next to each other based on storage time. The DBS samples stored at +4 °C for 40 years (or more precisely for 6 years at RT followed by 34 years at +4 °C) differed significantly from the 30-year-old samples (*p* value < 0.001). A few other comparisons also demonstrated significant differences between nearby groups but with lower significance (*p* value < 0.05). Based on this observation the samples stored at RT/+4 °C for 40 years were excluded from further analysis. Also, fresh samples stored at −24 °C were excluded from further analysis because they exhibited a lower group average compared with the samples stored for 0.5 and 10 years. Hence, for the analysis of relative abundances of individual proteins between groups the samples stored for 10, 20 and 30 years were compared with the samples stored for 0.5 years. This was done both for samples stored at +4 °C and −24 °C.

**Fig. 3.**
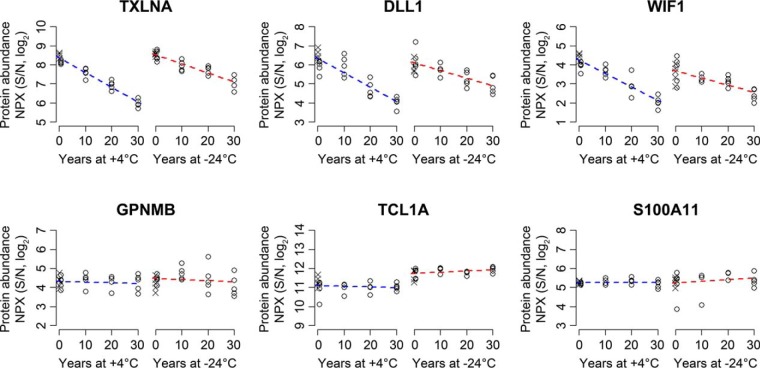
**Abundance of proteins measured in DBS stored between 0 and 30 years at +4 °C or −24 °C.** DBS samples from five individuals were analyzed for each condition. NPX values above LOD (protein abundance above background on a log_2_ scale) for six proteins were visualized in scatterplots together with linear regression models (dashed lines), demonstrating effects of storage time on measured protein levels. The six proteins highlighted in the figure are alpha-taxilin (TXLNA), delta-like protein 1 (DLL1), Wnt inhibitory factor 1 (WIF1), transmembrane glycoprotein NMB (GPNMB), T-cell leukemia/lymphoma protein 1A (TCL1A) and protein S100-A11 (S100A11). Samples stored for 0 years are represented with crosses and samples stored for 0.5, 10, 20, and 30 years are represented with open circles. The measured levels of the three proteins in the top row clearly decreased with increasing storage time, while no significant effect was demonstrated for the three proteins in the bottom row. The patterns were similar for samples stored at +4 °C and −24 °C but recorded levels of the three unstable proteins decreased faster at the higher storage temperature.

To enable comparisons of individual proteins, linear regression models were calculated for each of the proteins measured from DBS samples stored for up to 30 years at +4 °C (supplemental Fig. S3) or −24 °C (supplemental Fig. S4). The models were based on storage time in years and protein abundances as NPX values on a log_2_ scale. The purpose of the modeling was to identify proteins whose detectability was significantly affected by storage at the two temperature conditions. The degree of variation explained and the probability of the models were examined by ANOVA tests (supplemental Table S5). Clear differences regarding maintained detectability were observed among the proteins during long term storage both for DBS stored at +4 °C and −24 °C (Fig. 3). Some proteins were not detectably affected by storage time (*e.g.* GPNMB, TCL1A, and S100A11), while levels of others clearly decreased over time (*e.g.* TXLNA, DLL1 and WIF1).

**Fig. 4.**
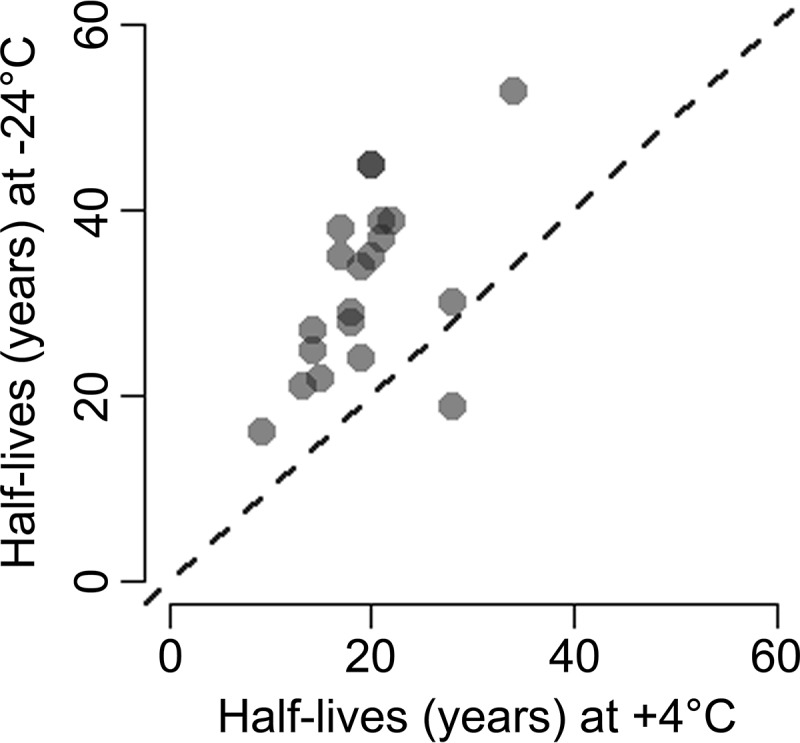
**Half-lives in years visualized in a scatter plot for the 20 investigated proteins that decreased significantly with increasing time of storage of DBS samples at +4 °C and −24 °C.** The half-lives were calculated from linear models based on NPX (protein abundance on a log_2_ scale) values and storage time. One half-life is the time required for the average level of a protein measurement to decrease by one NPX unit. 19 out of the 20 proteins exhibited longer half-lives for samples stored at −24 °C. The dashed line represents equal half-life for samples stored at +4 °C and −24 °C (x = y).

Models predicting significantly decreased protein levels with increasing storage time were evaluated for testing of multiple hypotheses (Bonferroni, *p* < 0.05/92). Sixty-one and 22 out of the 92 models evaluated showed significant decreases for samples stored at +4 °C and −24 °C, respectively, (supplemental Table S5). Twenty out of the 22 significantly decreasing proteins at −24 °C exhibited decreased levels also at +4 °C. The half-life of detectability for these 20 proteins was calculated based on their linear models (Fig. 4). Nineteen out of the 20 proteins exhibited longer half-life at −24 °C than at +4 °C. The most significant decrease was observed for alpha-taxilin (TXLNA) stored at +4 °C (*p* value = 1.5 × 10^−15^) or at −24 °C (*p* value = 1.5 × 10^−10^). The models explained 95 and 84% of the variation in TXLNA levels for samples stored at +4 °C and −24 °C, respectively.

**Fig. 5.**
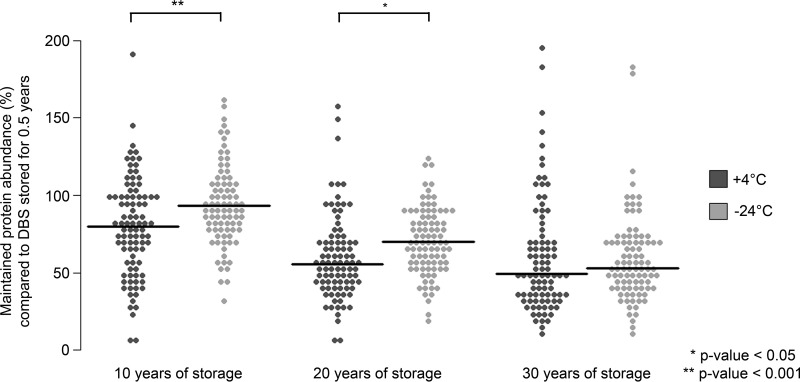
**Bee swarm plots visualizing the distributions of relative maintained signals for the 92 proteins analyzed compared with samples stored for 0.5 year at +4 °C and −24 °C.** The DBS samples were stored at +4 °C (dark gray) or −24 °C (light gray). The median value for each protein among the five individuals analyzed in each group was used for the analysis. The black lines in the distributions represent median values across all proteins. The median maintained signal across all investigated proteins are 80, 55 and 49% for the samples stored for 10, 20, and 30 years at +4 °C, respectively, and 93, 70 and 53% for the samples stored for 10, 20, and 30 years at −24 °C, respectively. Independent two group t-tests were used to evaluate statistical significances between the groups as indicated by asterisks. Outliers are not shown for visualization purposes but were included in the evaluation of statistical significances. The outliers were in total 12 measurements in the range between 200 and 420% of maintained signals. 8 out of the 12 outliers were in the group of samples stored at −24 °C for 10 years. The plot area was constrained between zero and 200% of relative signal.

To further compare samples stored at +4 °C or at −24 °C abundances of individual proteins from samples stored 10, 20 and 30 years were compared with levels of the proteins in samples stored for 0.5 year. This analysis was performed separately for samples stored at +4 °C and −24 °C, resulting in distributions of the proportions of maintained signals for all proteins measured. NPX (log_2_ scale) values were transformed to linear scale before calculating relative abundances. The results were visualized in a bee swarm plot, demonstrating decreasing trends of relative protein levels with increasing storage time both for samples stored at +4 °C and −24 °C (Fig. 5). After 10 years of storage at +4 °C or −24 °C the median of protein abundance remained at 80 and 93%, respectively, of the levels in samples that had been stored for 0.5 years. Independent two-group t-tests were performed to evaluate statistical significances of decreases of protein levels in samples stored at +4 °C or −24 °C. Statistically significant differences in protein detectability for samples stored at the two temperatures (*p* value < 0.05) were demonstrated for groups stored for 10 and 20 years, but no significant difference was observed for samples stored for 30 years.

##### Stability of Proteins in Plasma and Serum Samples Stored for up to 45 Years at −70°C

Correlation of protein abundances as measured by PEA taking into account the individual ages and the sample source (serum or plasma) for the sampled individuals was also investigated in 50 fresh-frozen samples. Age was observed to influence 58 out of the 92 proteins analyzed when evaluating the nominally statistically significant (*p* < 0.05) linear models in control plasma and serum samples (supplemental Table S6). This effect by donor age on protein levels is consistent with previous observations (11, 12). Nominally statistically significant differences (*p* < 0.05) were also demonstrated for 58 out of the 92 proteins when comparing plasma and serum samples for the same sampled individuals. Abundances of proteins measured in the ULSAM biobank with liquid plasma or serum samples that had been stored at −70 °C were individually normalized for effects of age of the individuals and sample type (serum or plasma) using the models from the previous step. Linear models predicting the relationship between NPX values above LOD and storage time were fitted to the normalized ULSAM data (supplemental Fig. S5). ANOVA tests were performed on the linear models. The trend for reduced detectability of the proteins was less apparent for the wet ULSAM samples stored at −70 °C than for the DBS samples. Only 5 out of the 92 linear models analyzed were significant when testing for multiple hypotheses (Bonferroni correction, *p* < 0.05/92) (supplemental Table S6). Not surprisingly, the fitted models for ULSAM samples collected from adult men and stored wet at −70 °C indicated greater variability among the sampled individuals compared with the DBS samples from newborns (on average 3.9 and 37% of the total variation was explained by storage time for the ULSAM samples and the DBS samples, respectively), limiting our ability to evaluate the effects of long term storage under the different storage conditions. Levels of three proteins (S100A4, HGF, and ADAM 8) decreased significantly both in wet samples and DBS stored at +4 °C, whereas no proteins were significantly decreased in both wet samples and DBS stored at −24 °C.

## DISCUSSION

Much effort is being devoted to finding genetic association to disease by genome wide association studies (GWAS). The work has generated invaluable insights into risk factors for complex disease, but the diagnostic usefulness has proven limited (13–15). There is now a rapid shift in focus in favor of searching for non-genetic biomarkers with dynamic properties, such as proteins, transcripts, and metabolites (16–19). Dynamic biomarkers are not constant between individuals and over time. Hence, individual baselines are preferably measured to accurately monitor relative fluctuations that may indicate emerging disease. In particular, there is considerable hope that measurements of blood proteins might yield information of value to diagnose disease processes anywhere in the body, or to select and monitor therapies, but few validated protein diagnostic markers have emerged in recent years. Nonetheless, improved techniques for sensitive, parallel protein analysis is now becoming available and rapid further development can be anticipated. It is therefore timely to consider how biobanks, suitable for the task may be constructed. Unlike GWAS where single samples collected either before or after onset of disease suffice, discovery of blood protein biomarkers should ideally be based on multiple samples collected over time for each individual, in samples that maintain the dynamic protein pattern during storage. Dried spots of blood or plasma are inexpensive to collect and to maintain, but three important aspects must be considered concerning their suitability as biobanks for protein analyses: (1) what happens with the detectability of proteins upon drying, (2) how are protein levels maintained upon long term storage, and (3) how does the long term stability compare between samples stored dried or wet. The present study addresses each of these questions, but it is important to note that it does so from the perspective of protein detection by pairs of antibodies in PEA tests. It is possible that results depend on the specific antibodies used or indeed whether antibodies are used at all or if trypsin digested peptides are analyzed by mass spectrometry to record protein levels, and the latter method is independent of the preservation of protein secondary structure.

The first consideration regarding the impact by drying and short-term storage is of relevance not only for biobanking but also for the rapidly expanding field of wellness monitoring, where samples may ideally be collected by the individuals themselves by a finger prick and sent dry by regular mail to a laboratory for analysis of protein levels (20, 21). Our investigation using a set of 92 protein assays demonstrates that detectability of proteins is very well preserved upon drying. We observed modest but consistent effects on measured levels by drying, motivating that dried samples should be compared with other dried samples and not directly to liquid ones. The correlation between wet and dried EDTA blood was on average 0.970, whereas the correlation between technical replicates was 0.999 both for wet and dried samples. Further studies may identify means to compensate for the slight effect of drying. Alternatively, the reproducible and stable effect of drying may be taken into account in order to compare fresh samples to standard curves prepared from dried controls. The considerable differences observed between protein levels in whole blood and plasma, whether in liquid or dried state, is an obvious consequence of the abundant presence of red and white cells in whole blood, contributing to the protein content.

The abundance of proteins that do not derive from the cellular fraction of blood is noticeably lower in wet blood compared with in wet plasma (Fig 1*C*, gray data points). This is explained by the fact that the 1 μl blood samples contained ∼0.5 μl cells and 0.5 μl plasma, or half the amount of plasma compared with the 1 μl plasma samples. Therefore ∼2-fold higher levels of plasma proteins, or 1 NPX, are expected in wet plasma samples compared with the levels in whole blood. The same effect is not seen for dried samples although 1.2 mm diameter disks were analyzed both for dried blood and dried plasma (Fig. 1*D*). This is probably a consequence of the circumstance that plasma spreads more than blood when applied to paper, resulting in more closely similar amounts of plasma proteins per punched out disks from dried plasma and dried blood.

Measurement of cellular proteins in DBS may permit diagnostically useful analyses of the cellular composition in blood samples, but direct comparison to plasma samples are difficult. Several approaches have been developed to separately collect cells and plasma from drops of blood, before they are dried on paper, and may further increase the value of biobanks of dried blood samples (22–27).

Protein distribution homogeneity across a DBS is important if many small portions are to be analyzed. Our investigation indicates slight, nominally significant increased levels of a subset of the analyzed proteins in samples collected from the edges compared with the middle of DBS, but the significance is lost upon correction for multiple hypothesis testing. Earlier studies have reported uneven distribution in DBS and explained these as so-called coffee-stain effects from the drying blood samples (28, 29). The concentration differences we observed do not in general exceed the technical variation between measurements from two equivalent DBS, and will have little impact on results.

Regarding the suitability for long-term storage of samples for protein analysis in the dried state our analysis of up to 30 years old dried and wet samples demonstrate that the majority of the proteins remain detectable over this period. For some proteins, no decreases could be detected even after 30 years of storage, but for several proteins a clear trend toward decreasing levels was observed with half-lives of some protein levels as low as 10 years. In dried samples stored at +4 °C or at −24 °C for 30 years, 61 and 22, respectively, of 92 investigated proteins were significantly decreased. The corresponding number for wet samples stored at −70 °C for 45 years was 5 out of the 92 proteins. The analysis is complicated by the fact that the different individuals sampled presumably have distinct protein levels, but the trend toward lower detected protein levels is nonetheless clear.

Differences in storage practices between the Swedish and the Danish newborn biobanks allowed a direct comparison of relative stabilities of protein levels over time, and we conclude that a storage temperature of −24 °C better preserves protein levels in dried blood samples than +4 °C (*e.g.* 80 and 93% of median protein abundance remained after 10 years of storage at +4 °C or at −24 °C, respectively). By inference it seems likely that a still lower storage temperature might have further extended the stability of the proteins. Here, DBS offer a strong advantage over liquid samples because of the very limited space needed for their storage in low-temperature freezers.

Regarding the third and final aspect about differences in protein stability between samples stored frozen either as liquids or dried on paper several aspects limit the power of the comparison in our study; (1) the dried samples represent whole blood while the frozen liquid samples were plasma or serum. (2) The dried samples were stored at +4 °C or −24 °C whereas the wet samples were stored at −70 °C. (3) The dried samples were obtained from newborns but the wet samples were from 50 to 90 year old adult men, and there was an inverse correlation between storage time and the age of the sampled men. (4) It may also be relevant that in contrast to the dried blood samples liquid plasma samples stored frozen included EDTA serving to chelate divalent cations, required for some enzymatic reactions. To make the comparison as reliable as possible the frozen plasma or serum samples were corrected for the variation in protein levels caused by age of individuals and type of sample by analysis of separate controls. With these factors in mind we conclude that proteins are better preserved in wet plasma and serum samples stored at −70 °C than as DBS stored at either +4 °C or at −24 °C. Over a period of 45 years only 5% of the analyzed proteins were significantly decreased when stored wet at −70 °C, compared with 66 and 24% for samples stored dried at +4 °C or at −24 °C, respectively.

An overall conclusion of our study is that proteins retain full detectability to a surprising extent in dried samples, although minor, consistent deviations of protein levels are observed upon drying. Regarding the possibility to preserve samples over longer times, either wet or dry, our experiments do not allow a direct, unambiguous comparison, but we find that both classes of samples reveal deterioration of levels of some of the proteins over time, as measured by antibody-based PEA tests. Lower storage temperatures are preferable, and it is possible that long term stability of protein levels at a given temperature are quite similar for samples stored dried or wet.

Typical reasons for protein degradation in aqueous solution are aggregation and chemical degradation. Aggregation can be triggered by different acute stresses, *e.g.* exposure to interfaces, changes in pH, freeze-thawing and elevated temperatures (30). The most prevalent types of chemical degradation are due to oxidation and hydrolysis (31–33). Several methods to stabilize proteins as powders upon drying have been developed, *e.g.* lyophilization, spray drying, pulverization, precipitation, supercritical fluid precipitation, spray-freeze drying, fluidized-bed spray coating, and emulsion precipitation (34). Another known feature is that enzymes can be stabilized by addition of different sugars such as trehalose to samples dehydrated by air-drying (35). It may prove possible to further improve the long-term stability of proteins in samples dried on paper by counteracting some of the processes leading to their deterioration.

The results of our study are encouraging as they suggest an inexpensive means to collect, transport, and store biobanked samples as spots of either whole blood or possibly separated plasma and cells, dried on paper. Our main findings were that (1) the act of drying only slightly influenced detection of blood proteins with an average correlation of 0.970 between levels in the wet and dried state, and the effect was reproducible with a correlation of 0.999 between duplicate samples of either wet or dry samples, (2) detection of some proteins was not significantly affected by storage over the full range of three decades. This was true for 34 and 76% of the analyzed proteins in dried samples stored at +4 °C and −24 °C, respectively, while levels of others decreased slowly during storage with half-lives in the range of 10 to 50 years, (3) detectability of proteins was more affected in dried samples stored at +4 °C compared with at −24 °C, for which the median protein abundance remained at 80 and 93% of starting levels after 10 years of storage at +4 °C and −24 °C, respectively, and (4) protein levels appeared more stable in liquid samples stored at −70 °C compared with DBS stored at −24 °C. Only 5% of the analyzed proteins significantly decreased when stored wet at −70 °C for 45 years compared with 24% for proteins in dried samples stored at −24 °C for 30 years, suggesting that temperature of storage is a major factor. Combined with improved means to measure hundreds and thousands of protein, such biobanks could prove of great medical value by enabling discovery of biomarkers for early detection of disease, as well as for patient management with important beneficial consequences for healthcare.

## Supplementary Material

Supplemental Data

## Footnotes

^1^ The abbreviations used are:

DBS: Dried blood spot
ADAM 8: Disintegrin and metalloproteinase domain-containing protein 8
ANOVA: Analysis of variance
DLL1: Delta-like protein 1
DPS: Dried plasma spot
EDTA: Ethylenediaminetetraacetic acid
GPNMB: Transmembrane glycoprotein NMB
GWAS: Genome wide association studies
HGF: Hepatocyte growth factor
LOD: Limit of detection
NPX: Normalized protein expression
PEA: Proximity extension assay
RT: Room temperature
S100A4: Protein S100-A4
S100A11: Protein S100-A11
TCL1A: T-cell leukaemia/lymphoma protein 1A
TXLNA: Alpha-taxilin
ULSAM: Uppsala longitudinal study of adult men
WIF1: Wnt inhibitory factor.
