# Supplementary material for: Stability of Proteins in Dried Blood Spot Biobanks
Source: Mol Cell Proteomics. 2017 May 13;16(7):1286–96. doi: 10.1074/mcp.RA117.000015 (PMC5500761; doi:10.1074/mcp.RA117.000015)
Supplement: Supplemental Data [file supp_16_7_1286__index.html]

Stability of Proteins in Dried Blood Spot Biobanks — Stability of Proteins in Dried Blood Spot Biobanks — Supplemental Data 

# Stability of Proteins in Dried Blood Spot Biobanks

## Supplemental Data

- Supplemental data - Supplemental figures and tables
