# Supplementary material for: Stability of Proteins in Dried Blood Spot Biobanks
Source: Mol Cell Proteomics. 2017 May 13;16(7):1286–96. doi: 10.1074/mcp.RA117.000015 (PMC5500761; doi:10.1074/mcp.RA117.000015)
Supplement: Supplemental Data [file supp_RA117.000015_4536_1_supp_927_tqjtkm.pdf]

Stability of Proteins in Dried Blood Spot Biobanks

Johan Björkesten, Stefan Enroth, Qiujin Shen, Lotta Wik, David M. Hougaard, Arieh S. Cohen, Lene Sörensen, Vilmantas Giedraitis, Martin Ingelsson, Anders Larsson, Masood Kamali Moghaddam and Ulf Landegren*

*To whom the correspondence should be addressed: ulf.landegren@igp.uu.se

Supplemental Figures and Tables

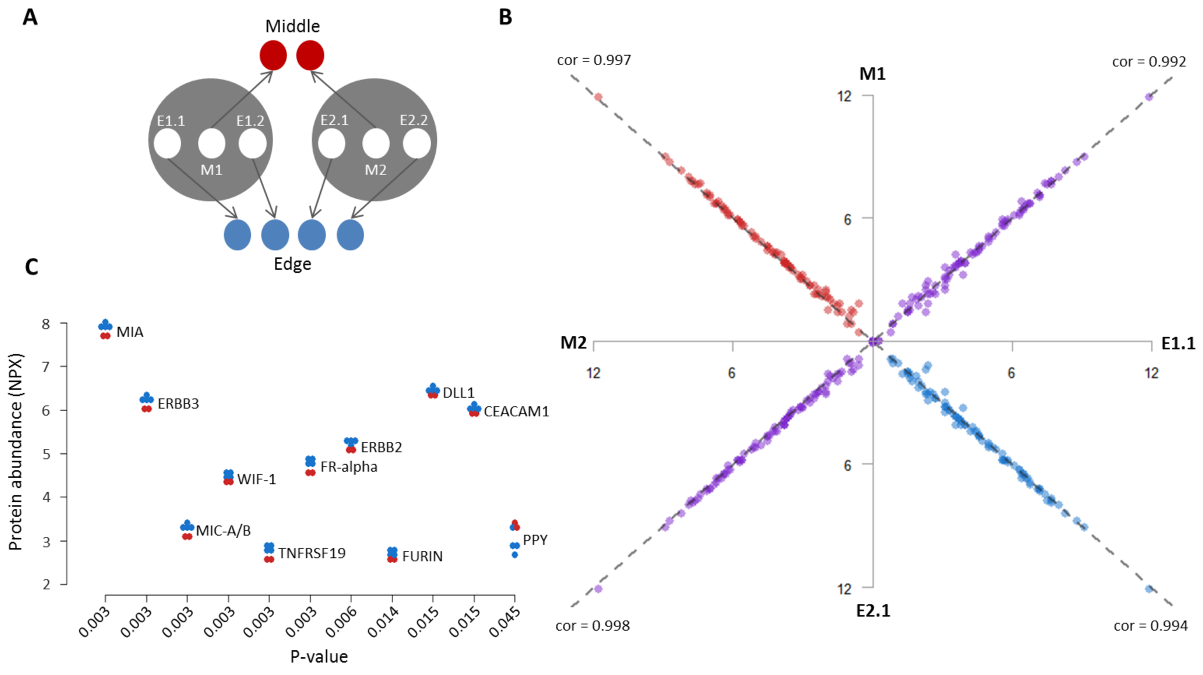

Supplemental Figure S1. Evaluation of DBS homogeneity by analysis of protein abundance by analysis of disks punched out from the middle and edges of two equivalent DBS. A) Schematic figure of the analyzed samples. Two equivalent DBS (grey) were prepared and three 1.2 mm diameter disks (two from the edge and one from the middle) were punched out from each of them. Abundances of 92 proteins were measured from each disk by PEA. The disks were grouped as edge or middle for statistical analysis. B) Relations between replicates evaluating potential differences between samples from the middle and edge of DBS and two equivalent DBS are visualized in a scatterplot, containing four correlations. Correlation between samples from the middle of two equivalent DBS (red) were 0.997. Correlation between samples from the edge of two equivalent DBS (blue) were 0.994. Correlations between samples from the edge and middle of each of the two DBS (purple) were 0.992 and 0.998, respectively. The dashed grey lines corresponds to the equivalence between compared samples (x = y). Individual levels of LOD (log_2-scale) have been subtracted from NPX values (log_2-scale) for each protein. Any resulting negative values (signals below LOD) were set to zero. C) Independent 2-group t-tests were performed for each of the 92 proteins to evaluate potential differences between the edge and middle groups. 11 out of the 92 proteins analyzed exhibited nominally significant differences (p-value < 0.05), however, none of the analyzed proteins were significantly different when correcting for multiple hypothesis testing (Bonferroni, p-value < 0.05/92). Individual protein abundances (NPX values on log_2-scale), measured in samples from the middle (red) and edge (blue) of DBS for the 11 nominally significantly changed proteins, was

visualized as bee swarm plots. The bee swarm representations are sorted from left to right based on increasing p-value. The short name of the respective protein is shown next to the corresponding data points.

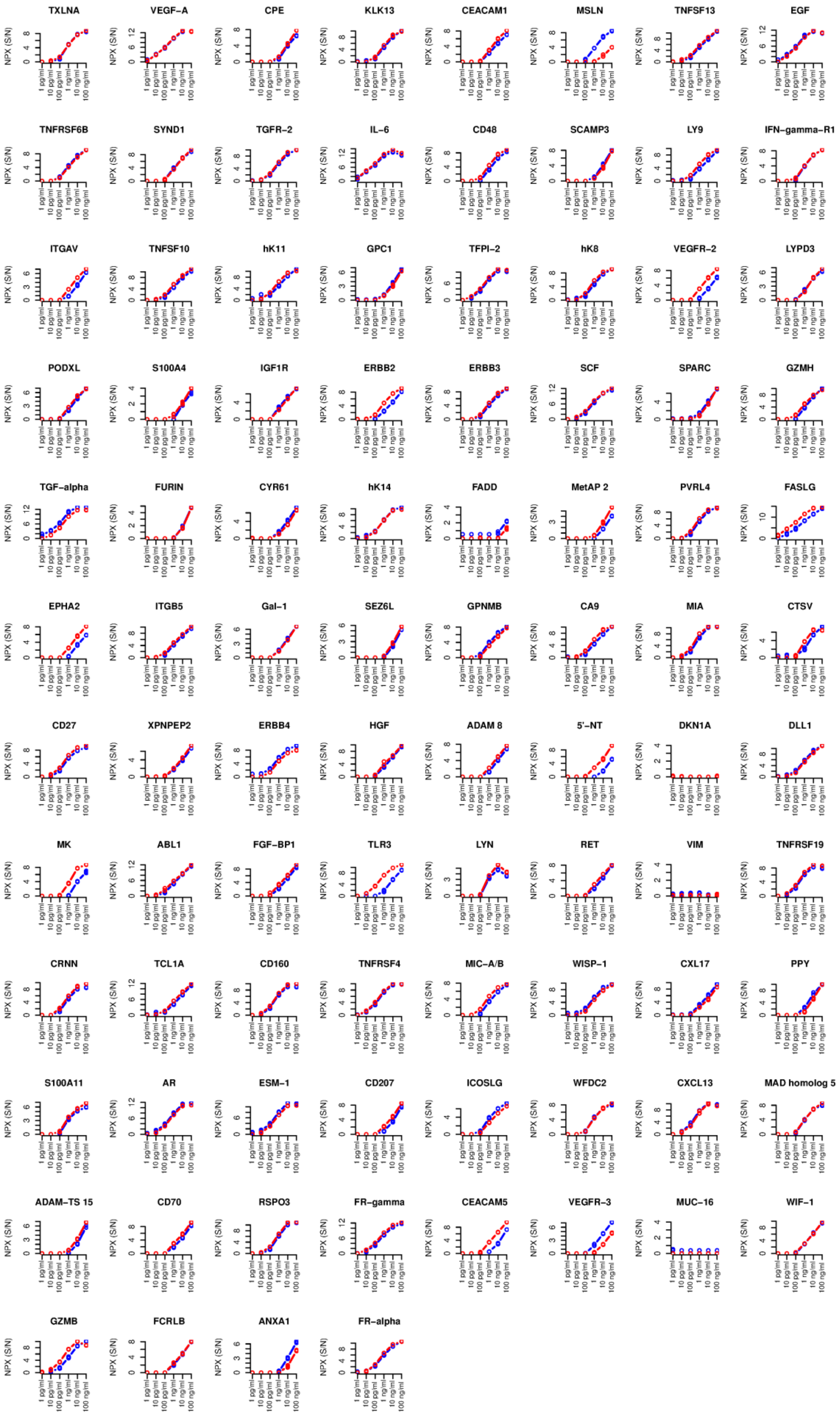
TXLNA
VEGF–A
CPE
KLK13
CEACAM1
MSLN
TNFSF13
EGF
TNFRSF6B
SYND1
TGFR–2
IL–6
CD48
SCAMP3
LY9
IFN–gamma–R1
ITGAV
TNFSF10
hK11
GPC1
TFPI–2
hK8
VEGFR–2
LYPD3
PODXL
S100A4
IGF1R
ERBB2
ERBB3
SCF
SPARC
GZMH
TGF–alpha
FURIN
CYR61
hK14
FADD
MetAP 2
PVRL4
FASLG
EPHA2
ITGB5
Gal–1
SEZ6L
GPNMB
CA9
MIA
CTSV
CD27
XPNPEP2
ERBB4
HGF
ADAM 8
5'–NT
DKN1A
DLL1
MK
ABL1
FGF–BP1
TLR3
LYN
RET
VIM
TNFRSF19
CRNN
TCL1A
CD160
TNFRSF4
MIC–A/B
WISP–1
CXL17
PPY
S100A11
AR
ESM–1
CD207
ICOSLG
WFDC2
CXCL13
MAD homolog 5
ADAM–TS 15
CD70
RSPO3
FR–gamma
CEACAM5
VEGFR–3
MUC–16
WIF–1
GZMB
FCRLB
ANXA1
FR–alpha
NPX (S/N)
1 pg/ml
10 pg/ml
100 pg/ml
1 ng/ml
10 ng/ml
100 ng/ml

Supplemental Figure S2. Individual plots of NPX-values above LOD for 10-fold dilution series (1 pg/ml - 100 ng/ml) for a pool of 92 measured recombinant proteins in purified form. Both wet (red) and dried (blue) samples were analyzed. All data points from technical triplicates are plotted in the figure although data largely overlap due to the high precision of PEA multiplex measurements. The lines connecting the data points were drawn between the mean values of the technical triplicates for each concentration. The Proseek multiplex panel used for the analysis was Oncology I v2.

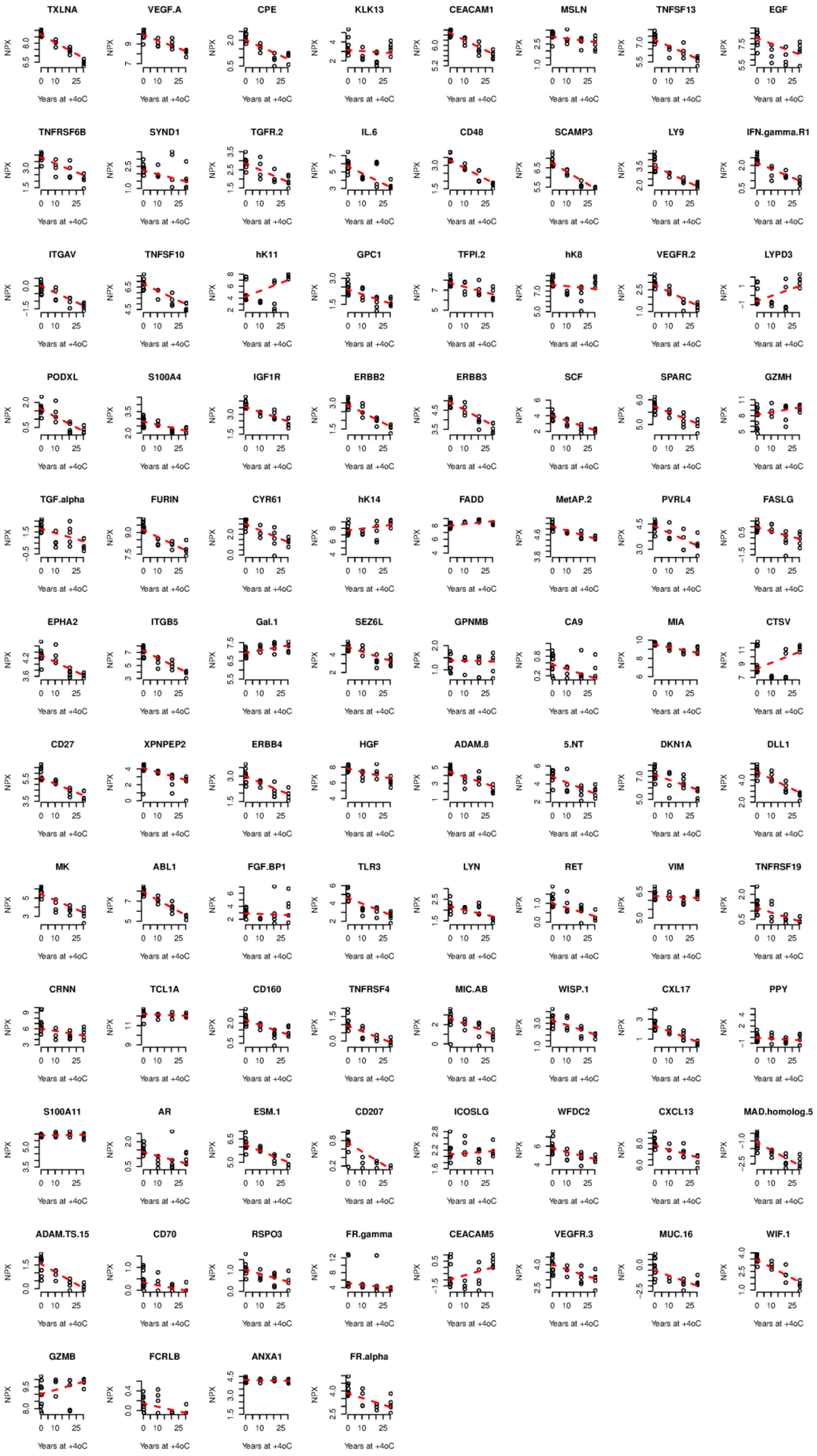
TXLNA
VEGF.A
CPE
KLK13
CEACAM1
MSLN
TNFSF13
EGF
TNFRSF6B
SYND1
TGFR.2
IL.6
CD48
SCAMP3
LY9
IFN.gamma.R1
ITGAV
TNFSF10
hK11
GPC1
TFPI.2
hK8
VEGFR.2
LYPD3
PODXL
S100A4
IGF1R
ERBB2
ERBB3
SCF
SPARC
GZMH
TGF.alpha
FURIN
CYR61
hK14
FADD
MetAP.2
PVRL4
FASLG
EPHA2
ITGB5
Gal.1
SEZ6L
GPNMB
CA9
MIA
CTSV
CD27
XPNPEP2
ERBB4
HGF
ADAM.8
5.NT
DKN1A
DLL1
MK
ABL1
FGF.BP1
TLR3
LYN
RET
VIM
TNFRSF19
CRNN
TCL1A
CD160
TNFRSF4
MIC.AB
WISP.1
CXL17
PPY
S100A11
AR
ESM.1
CD207
ICOSLG
WFDC2
CXCL13
MAD.homolog.5
ADAM.TS.15
CD70
RSPO3
FR.gamma
CEACAM5
VEGFR.3
MUC.16
WIF.1
GZMB
FCRLB
ANXA1
FR.alpha
NPX
Years at +4oC

Supplemental Figure S3. Individual linear regression models (dashed red lines) for all 92 proteins measured in DBS samples stored between 0 and 30 years at +4°C. The models were based on storage time in years and protein abundance as NPX values on a $\log_2$ scale.

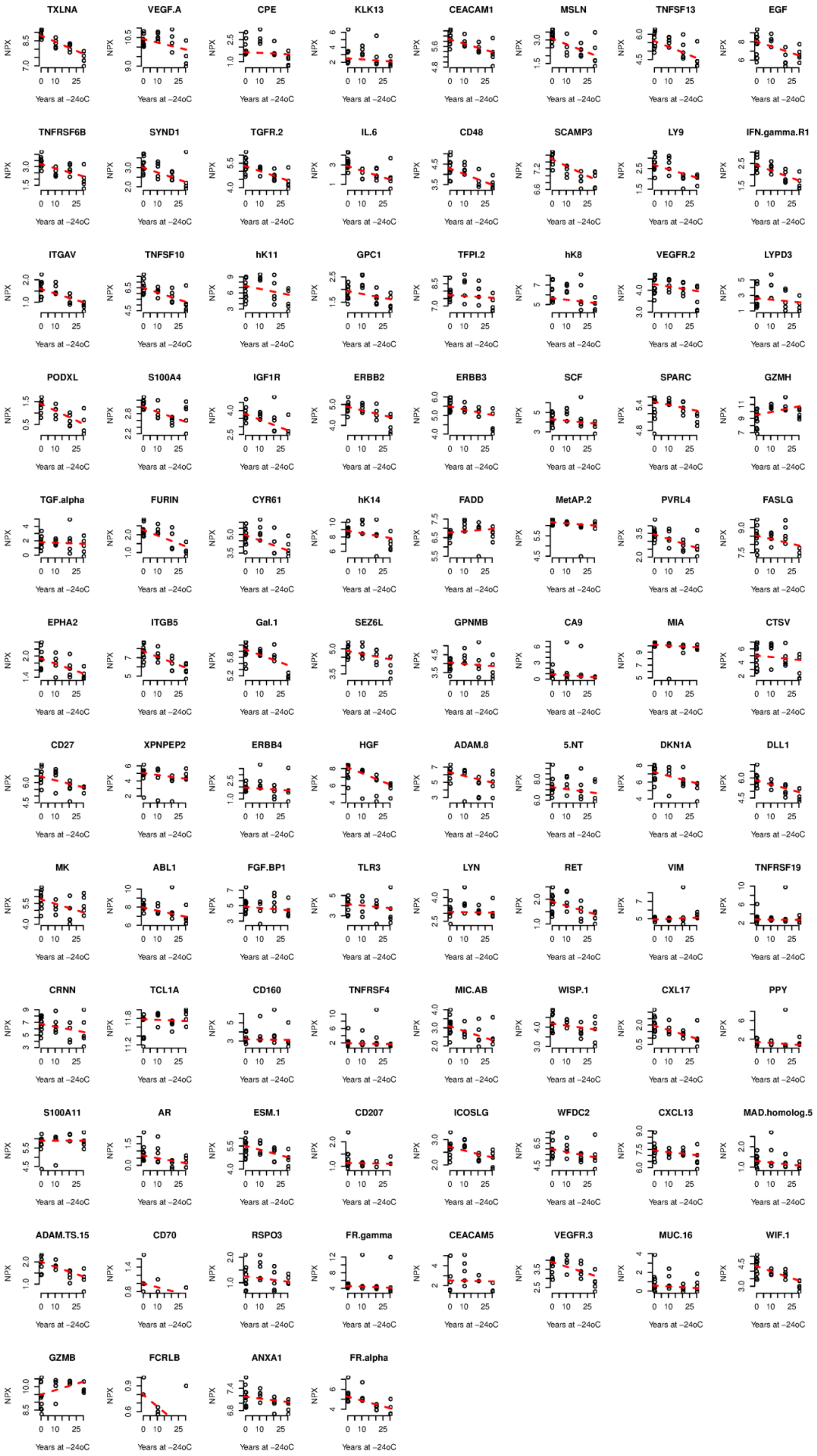
TXLNA
VEGF.A
CPE
KLK13
CEACAM1
MSLN
TNFSF13
EGF
TNFRSF6B
SYND1
TGFR.2
IL.6
CD48
SCAMP3
LY9
IFN.gamma.R1
ITGAV
TNFSF10
hK11
GPC1
TFPI.2
hK8
VEGFR.2
LYPD3
PODXL
S100A4
IGF1R
ERBB2
ERBB3
SCF
SPARC
GZMH
TGF.alpha
FURIN
CYR61
hK14
FADD
MetAP.2
PVRL4
FASLG
EPHA2
ITGB5
Gal.1
SEZ6L
GPNMB
CA9
MIA
CTSV
CD27
XPNPEP2
ERBB4
HGF
ADAM.8
5.NT
DKN1A
DLL1
MK
ABL1
FGF.BP1
TLR3
LYN
RET
VIM
TNFRSF19
CRNN
TCL1A
CD160
TNFRSF4
MIC.AB
WISP.1
CXL17
PPY
S100A11
AR
ESM.1
CD207
ICOSLG
WFDC2
CXCL13
MAD.homolog.5
ADAM.TS.15
CD70
RSPO3
FR.gamma
CEACAM5
VEGFR.3
MUC.16
WIF.1
GZMB
FCRLB
ANXA1
FR.alpha
NPX
Years at -24oC

Supplemental Figure S4. Individual linear regression models (dashed red lines) for all 92 proteins measured from DBS samples stored between 0 and 30 years at -24°C. The models were based on storage time in years and protein abundance as NPX values on a log_2 scale.

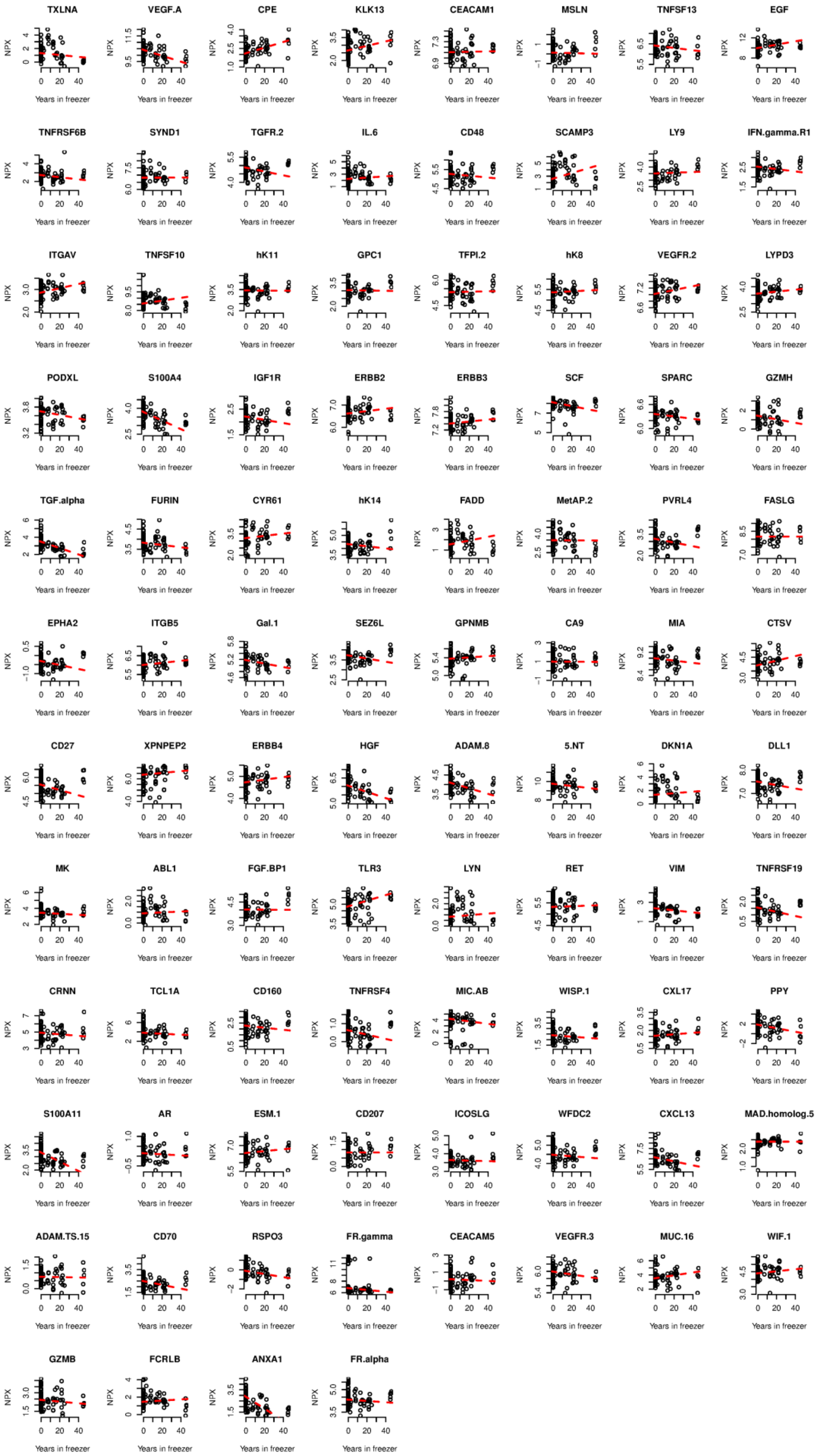
TXLNA
VEGF.A
CPE
KLK13
CEACAM1
MSLN
TNFSF13
EGF
TNFRSF6B
SYND1
TGFR.2
IL.6
CD48
SCAMP3
LY9
IFN.gamma.R1
ITGAV
TNFSF10
hK11
GPC1
TFPI.2
hK8
VEGFR.2
LYPD3
PODXL
S100A4
IGF1R
ERBB2
ERBB3
SCF
SPARC
GZMH
TGF.alpha
FURIN
CYR61
hK14
FADD
MetAP.2
PVRL4
FASLG
EPHA2
ITGB5
Gal.1
SEZ6L
GPNMB
CA9
MIA
CTSV
CD27
XPNPEP2
ERBB4
HGF
ADAM.8
5.NT
DKN1A
DLL1
MK
ABL1
FGF.BP1
TLR3
LYN
RET
VIM
TNFRSF19
CRNN
TCL1A
CD160
TNFRSF4
MIC.AB
WISP.1
CXL17
PPY
S100A11
AR
ESM.1
CD207
ICOSLG
WFDC2
CXCL13
MAD.homolog.5
ADAM.TS.15
CD70
RSPO3
FR.gamma
CEACAM5
VEGFR.3
MUC.16
WIF.1
GZMB
FCRLB
ANXA1
FR.alpha
NPX
Years in freezer

Supplemental Figure S5. Individual linear regression models (dashed red lines) for all 92 proteins measured from liquid ULSAM plasma or serum samples stored between 0 and 45 years at -70°C. The models were based on storage time in years and protein abundance as NPX values on a $\log_2$ scale. Protein abundance was normalized for significant effects of age of individuals and sample type (plasma/serum).

Supplemental Table S1. Protein assays in the Proseek Multiplex Oncology II panel. See the Olink website for additional information (www.olink.com).

**Oncology II**		
**Long Name**	**Short Name**	**Uniprot**
Alpha-taxilin	TXLNA	P40222
Vascular endothelial growth factor A	VEGF-A	P15692
Carboxypeptidase E	CPE	P16870
Incubation Control 1	Inc Ctrl 1	-
Kallikrein-13	KLK13	Q9UKR3
Carcinoembryonic antigen-related cell adhesion molecule 1	CEACAM1	P13688
Mesothelin	MSLN	Q13421
Tumor necrosis factor ligand superfamily member 13	TNFSF13	O75888
Pro-epidermal growth factor	EGF	P01133
Tumor necrosis factor receptor superfamily member 6B	TNFRSF6B	O95407
Syndecan-1	SYND1	P18827
TGF-beta receptor type-2	TGFR-2	P37173
Interleukin-6	IL-6	P05231
CD48 antigen	CD48	P09326
Secretory carrier-associated membrane protein 3	SCAMP3	O14828
T-lymphocyte surface antigen Ly-9	LY9	Q9HBG7
Interferon gamma receptor 1	IFN-gamma-R1	P15260
Integrin alpha-V	ITGAV	P06756
Extension Control	Ext Ctrl	-
TNF-related apoptosis-inducing ligand	TRAIL	P50591
Kallikrein-11	hK11	Q9UBX7
Glypican-1	GPC1	P35052
Tissue factor pathway inhibitor 2	TFPI-2	P48307
Kallikrein-8	hK8	O60259
Vascular endothelial growth factor receptor 2	VEGFR-2	P35968
Ly6/PLAUR domain-containing protein 3	LYPD3	O95274
Podocalyxin	PODXL	O00592
Protein S100-A4	S100A4	P26447
Insulin-like growth factor 1 receptor	IGF1R	P08069
Receptor tyrosine-protein kinase erbB-2	ERBB2	P04626
Receptor tyrosine-protein kinase erbB-3	ERBB3	P21860
Stem cell factor	SCF	P21583
SPARC	SPARC	P09486

Granzyme H	GZMH	P20718
Transforming growth factor alpha	TGF-alpha	P01135
Furin	FURIN	P09958
Protein CYR61	CYR61	O00622
Kallikrein-14	hK14	Q9P0G3
FAS-associated death domain protein	FADD	Q13158
Methionine aminopeptidase 2	MetAP 2	P50579
Nectin-4	PVRL4	Q96NY8
Tumor necrosis factor ligand superfamily member 6	FASLG	P48023
Ephrin type-A receptor 2	EPHA2	P29317
Integrin beta-5	ITGB5	P18084
Galectin-1	Gal-1	P09382
Incubation Control 2	Inc Ctrl 2	-
Detection Control	Det Ctrl	-
Seizure 6-like protein	SEZ6L	Q9BYH1
Transmembrane glycoprotein NMB	GPNMB	Q14956
Carbonic anhydrase 9	CA9	Q16790
Melanoma-derived growth regulatory protein	MIA	Q16674
Cathepsin L2	CTSV	O60911
CD27 antigen	CD27	P26842
Xaa-Pro aminopeptidase 2	XPNPEP2	O43895
Receptor tyrosine-protein kinase erbB-4	ERBB4	Q15303
Hepatocyte growth factor	HGF	P14210
Disintegrin and metalloproteinase domain-containing protein 8	ADAM 8	P78325
5'-nucleotidase	5'-NT	P21589
Cyclin-dependent kinase inhibitor 1	DKN1A	P38936
Delta-like protein 1	DLL1	O00548
Midkine	MK	P21741
Tyrosine-protein kinase ABL1	ABL1	P00519
Fibroblast growth factor-binding protein 1	FGF-BP1	Q14512
Toll-like receptor 3	TLR3	O15455
Tyrosine-protein kinase Lyn	LYN	P07948
Proto-oncogene tyrosine-protein kinase receptor Ret	RET	P07949
Vimentin	VIM	P08670
Tumor necrosis factor receptor superfamily member 19	TNFRSF19	Q9NS68
Cornulin	CRNN	Q9UBG3
T-cell leukemia / lymphoma protein 1A	TCL1A	P56279
CD160 antigen	CD160	O95971
Tumor necrosis factor receptor superfamily member 4	TNFRSF4	P43489
MHC class I polypeptide-related sequence A and B	MIC-A/B	Q29983 / Q29980
WNT1-inducible-signaling pathway protein 1	WISP-1	O95388
VEGF-co regulated chemokine 1	CXL17	Q6UXB2
Pancreatic prohormone	PPY	P01298
Protein S100-A11	S100A11	P31949
Amphiregulin	AR	P15514

Endothelial cell-specific molecule 1	ESM-1	Q9NQ30
C-type lectin domain family 4 member K	CD207	Q9UJ71
ICOS ligand	ICOSLG	O75144
WAP four-disulfide core domain protein 2	WFDC2	Q14508
C-X-C motif chemokine 13	CXCL13	O43927
Mothers against decapentaplegic homolog 5	MAD homolog 5	Q99717
A disintegrin and metalloproteinase with thrombospondin motifs 15	ADAM-TS 15	Q8TE58
CD70 antigen	CD70	P32970
R-spondin-3	RSPO3	Q9BXY4
Folate receptor gamma	FR-gamma	P41439
Carcinoembryonic antigen-related cell adhesion molecule 5	CEACAM5	P06731
Vascular endothelial growth factor receptor 3	VEGFR-3	P35916
Mucin-16	MUC-16	Q8WXI7
Wnt inhibitory factor 1	WIF-1	Q9Y5W5
Granzyme B	GZMB	P10144
Fc receptor-like B	FCRLB	Q6BAA4
Annexin A1	ANXA1	P04083
Folate receptor alpha	FR-alpha	P15328

Supplemental Table S2. Description of analyzed wet samples stored at -70°C in the ULSAM biobank and fresh control samples used for normalization purposes.

**Analyzed samples from the ULSAM biobank**				
**Name**	**Age of individual (years)**	**Storage time (years)**	**Sample type**	**Thawed?**
U-S1	51	46	Serum	NA
U-S2	50	46	Serum	NA
U-S3	50	45	Serum	NA
U-S4	50	45	Serum	NA
U-S5	50	45	Serum	NA
U-S11	72	25	Serum	Yes
U-S14	72	22	Serum	Probably
U-S15	71	22	Serum	Probably
U-S17	70	22	Serum	Probably
U-S19	70	21	Serum	Probably
U-P21	71	23	EDTA plasma	Yes
U-P23	71	23	EDTA plasma	Probably
U-P27	70	23	EDTA plasma	Probably
U-P28	70	22	EDTA plasma	Probably

**Fresh samples analyzed from the department of Clinical Chemistry at Uppsala University Hospital**			
**Name**	**Age of individual (years)**	**Age group (years)**	**Sample type**
F-P7	53	50	EDTA plasma
F-P10	55	50	EDTA plasma
F-P14	49	50	EDTA plasma
F-P22	54	50	EDTA plasma
F-P27	54	50	EDTA plasma
F-S7	53	50	serum
F-S10	55	50	serum
F-S14	49	50	serum
F-S22	54	50	serum
F-S27	54	50	serum
F-P6	57	60	EDTA plasma
F-P9	56	60	EDTA plasma
F-P11	60	60	EDTA plasma
F-P15	57	60	EDTA plasma

U-P30	70	21	EDTA plasma	Probably
U-S42	78	17	Serum	No
U-S43	77	17	Serum	No
U-S45	77	16	Serum	No
U-S48	76	15	Serum	No
U-S49	76	15	Serum	No
U-P51	83	13	EDTA plasma	Yes
U-P52	83	13	EDTA plasma	Yes
U-P53	83	13	EDTA plasma	Yes
U-P54	83	13	EDTA plasma	Yes
U-P55	82	13	EDTA plasma	Yes
U-P61	88	8	EDTA plasma	No
U-P62	87	8	EDTA plasma	No
U-P63	87	8	EDTA plasma	No
U-P64	87	7	EDTA plasma	No
U-P65	86	7	EDTA plasma	No
U-P71	94	3	EDTA plasma	No
U-P72	93	2	EDTA plasma	No
U-P73	92	2	EDTA plasma	No
U-P74	91	2	EDTA plasma	No
U-P75	91	2	EDTA plasma	No

F-P16	60	60	EDTA plasma
F-S6	57	60	serum
F-S9	56	60	serum
F-S11	60	60	serum
F-S15	57	60	serum
F-S16	60	60	serum
F-P2	74	70	EDTA plasma
F-P5	70	70	EDTA plasma
F-P13	72	70	EDTA plasma
F-P18	70	70	EDTA plasma
F-P24	72	70	EDTA plasma
F-S2	74	70	serum
F-S5	70	70	serum
F-S13	72	70	serum
F-S18	70	70	serum
F-S24	72	70	serum
F-P3	77	80	EDTA plasma
F-P4	75	80	EDTA plasma
F-P12	81	80	EDTA plasma
F-P19	79	80	EDTA plasma
F-P20	82	80	EDTA plasma
F-S3	77	80	serum
F-S4	75	80	serum
F-S12	81	80	serum
F-S19	79	80	serum
F-P1	86	90	EDTA plasma
F-P8	91	90	EDTA plasma
F-P25	86	90	EDTA plasma
F-P26	90	90	EDTA plasma
F-P35	86	90	EDTA plasma
F-S1	86	90	serum
F-S8	91	90	serum
F-S25	86	90	serum
F-S26	90	90	serum
F-S35	86	90	serum

Supplemental Table S3. Protein assays in the Proseek Multiplex Oncology I v2 panel. See the Olink website for additional information (www.olink.com).

**Oncology I v2**		
**Long Name**	**Short Name**	**Uniprot**
Interleukin-8	IL-8	P10145
Vascular endothelial growth factor A	VEGF-A	P15692
Adrenomedullin	AM	P35318
Incubation Control 1	Inc Ctrl 1	-

CD40 ligand	CD40-L	P29965
Growth/differentiation factor 15	GDF-15	Q99988
Placenta growth factor	PlGF	P49763
E-selectin	SELE	P16581
Macrophage colony-stimulating factor 1	CSF-1	P09603
Immunoglobulin-like transcript 3	ILT-3	Q8NHJ6
Ezrin	EZR	P15311
Interleukin-1 receptor antagonist protein	IL-1ra	P18510
Interleukin-6	IL-6	P05231
Cystatin-B	CSTB	P04080
Monocyte chemotactic protein 1	MCP-1	P13500
Kallikrein-6	KLK6	Q92876
TNF-related apoptosis-inducing ligand receptor 2	TRAIL-R2	O14763
Erythropoietin	EPO	P01588
Extension Control	Ext Ctrl	-
Latency-associated peptide transforming growth factor beta-1	LAP TGF-beta-1	P01137
Kallikrein-11	hK11	Q9UBX7
Angiopoietin-1 receptor	TIE2	Q02763
Tissue factor	TF	P13726
Tumor necrosis factor receptor 1	TNF-R1	P19438
Platelet-derived growth factor subunit B	PDGF subunit B	P01127
Parkinson disease protein 7	PARK7	Q99497
Interleukin-2	IL-2	P60568
C-X-C motif chemokine 11	CXCL11	O14625
Interleukin-12	IL-12	P29460; P29459
Vascular endothelial statin	VE-statin	Q9UHF1
Interleukin-7	IL-7	P13232
Stem cell factor	SCF	P21583
C-X-C motif chemokine 9	CXCL9	Q07325
Interleukin-6 receptor subunit alpha	IL-6RA	P08887
Tumor necrosis factor receptor 2	TNF-R2	P20333
Matrix metalloproteinase-1	MMP-1	P03956
FAS-associated death domain protein	FADD	Q13158
Tumor necrosis factor ligand superfamily member 14	TNFSF14	O43557
Prolactin	PRL	P01236
Furin	FUR	P09958
Growth hormone	GH	P01241
Fas antigen ligand	FasL	P48023
B-cell activating factor	BAFF	Q9Y275
Tumor necrosis factor receptor superfamily member 6	FAS	P25445
C-C motif chemokine 19	CCL19	Q99731
Incubation Control 2	Inc Ctrl 2	-
Detection Control	Det Ctrl	-
Tyrosine-protein phosphatase non-receptor type 22	PTPN22	Q9Y2R2
Extracellular matrix metalloproteinase inducer	EMMPRIN	P35613

Carbonic anhydrase IX	CAIX	Q16790
C-X-C motif chemokine 10	CXCL10	P02778
Epithelial cell adhesion molecule	Ep-CAM	P16422
Receptor tyrosine-protein kinase erbB-2	ErbB2/HER2	P04626
Receptor tyrosine-protein kinase erbB-3	ErbB3/HER3	P21860
Receptor tyrosine-protein kinase erbB-4	ErbB4/HER4	Q15303
Hepatocyte growth factor	HGF	P14210
Lipopolysaccharide-induced tumor necrosis factor-alpha factor	LITAF	Q99732
Myeloid differentiation primary response protein MyD88	MYD88	Q99836
Melanoma-derived growth regulatory protein	MIA	Q16674
C-X-C motif chemokine 5	CXCL5	P42830
Midkine	MK	P21741
Urokinase plasminogen activator surface receptor	U-PAR	Q03405
Tumor necrosis factor	TNF	P01375
ICOS ligand	ICOSLG	O75144
Cadherin-3	CDH3	P22223
Tyrosine-protein kinase Lyn	LYN	P07948
Fms-related tyrosine kinase 3 ligand	Flt3L	P49771
Vascular endothelial growth factor receptor 2	VEGFR-2	P35968
Heparin-binding EGF-like growth factor	HB-EGF	Q99075
Caspase-3	CASP-3	P42574
Early activation antigen CD69	CD69	Q07108
Tumor necrosis factor receptor superfamily member 4	TNFRSF4	P43489
Tartrate-resistant acid phosphatase type 5	TR-AP	P13686
Cyclin-dependent kinase inhibitor 1	CDKN1A	P38936
Regenerating islet-derived protein 4	REG-4	Q9BYZ8
Transforming growth factor alpha	TGF-alpha	P01135
Amphiregulin	AR	P15514
Interferon gamma	IFN-gamma	P01579
MHC class I polypeptide-related sequence A	MIC-A	Q29983
NF-kappa-B essential modulator	NEMO	Q9Y6K9
Vascular endothelial growth factor D	VEGF-D	O43915
Epididymal secretory protein E4	HE4	Q14508
C-X-C motif chemokine 13	CXCL13	O43927
Epidermal growth factor receptor	EGFR	P00533
Integrin alpha-1	ITGA1	P56199
Thrombopoietin	THPO	P40225
Eukaryotic translation initiation factor 4B	eIF-4B	P23588
Carcinoembryonic antigen	CEA	P06731
Vimentin	VIM	P08670
NT-3 growth factor receptor	NTRK3	Q16288
Ovarian cancer-related tumor marker CA 125	CA-125	Q8WXI7
Prostasin	PRSS8	Q16651
Follistatin	FS	P19883
Platelet endothelial cell adhesion molecule	PECAM-1	P16284

Interleukin-17 receptor B	IL-17RB	Q9NRM6
Folate receptor alpha	FR-alpha	P15328

Supplemental Table S4. The 20 proteins farthest away from the line of equivalence (most highly elevated) in EDTA blood compared to EDTA plasma. The proteins are sorted according to decreasing concentration differences between whole blood and plasma in wet samples. The table also reports the concentration differences for the same proteins in dried samples. The ratio of plasma to cells differs between the wet and dried samples, accounting for the difference of higher average ratios in dried compared to wet samples. The color scale corresponds to the colors of the protein measurements plotted in Fig. 1C and D.

Short name	UniProt	Fold Change (wet)	Fold Change (dried)	Color scale
CASP-3	P42574	163	700	
NEMO	Q9Y6K9	124	239	
PTPN22	Q9Y2R2	91	58	
CDKN1A	P38936	71	45	
CD69	Q07108	44	76	
MYD88	Q99836	40	58	
CD40-L	P29965	37	38	
eIF-4B	P23588	36	120	
FADD	Q13158	35	115	
CXCL11	O14625	30	30	
LITAF	Q99732	19	26	
VIM	P08670	18	71	
CSTB	P04080	11	39	
TNFSF14	O43557	11	10	
IL-8	P10145	9	11	
IL-1ra	P18510	8	11	
CXCL5	P42830	8	12	
EZR	P15311	6	18	
LYN	P07948	6	18	
MCP-1	P13500	3	5	

Supplemental Table S5. Parameter values from ANOVA tests of linear regression models calculated individually for NPX values ($\log_2$) for all 92 proteins measured from DBS stored for 0 to 30 years at +4°C or -24°C. **β** describes the slope of the linear model in the unit NPX per year. **VarExp** describes the estimated proportion of variation that is explained by the model. **P-val** describes the probability

of the model. **Signif** is set to 1 if the parameter p-val is below the limit for testing of multiple hypotheses (0.05/92) and β is negative.

	+4°C				-24°C			
**Protein**	**β**	**VarExp**	**p-val**	**Signif**	**β**	**VarExp**	**p-val**	**Signif**
TXLNA	-0.0758	0.9474	0.0000	1	-0.0469	0.8378	0.0000	1
VEGF A	-0.0559	0.7333	0.0000	1	-0.0267	0.3336	0.0025	0
CPE	-0.0427	0.7157	0.0000	1	-0.0237	0.2052	0.0230	0
KLK13	-0.0159	0.0503	0.2921	0	-0.0297	0.0837	0.1606	0
CEACAM1	-0.0292	0.8673	0.0000	1	-0.0188	0.4657	0.0002	1
MSLN	-0.0196	0.3181	0.0041	0	-0.0377	0.4261	0.0004	1
TNFSF13	-0.0559	0.7690	0.0000	1	-0.0357	0.4351	0.0003	1
EGF	-0.0507	0.3537	0.0022	0	-0.0502	0.2410	0.0127	0
TNFRSF6B	-0.0528	0.7019	0.0000	1	-0.0414	0.4847	0.0001	1
SYND1	-0.0204	0.1382	0.0736	0	-0.0272	0.3356	0.0024	0
TGFR 2	-0.0362	0.6417	0.0000	1	-0.0331	0.5049	0.0001	1
IL 6	-0.0731	0.4723	0.0002	1	-0.0483	0.3552	0.0017	0
CD48	-0.0594	0.8985	0.0000	1	-0.0284	0.4476	0.0003	1
SCAMP3	-0.0509	0.8880	0.0000	1	-0.0222	0.6497	0.0000	1
LY9	-0.0590	0.7650	0.0000	1	-0.0265	0.4443	0.0003	1
IFN gamma R1	-0.0525	0.8109	0.0000	1	-0.0291	0.6257	0.0000	1
ITGAV	-0.0447	0.7497	0.0000	1	-0.0254	0.5521	0.0000	1
TNFSF10	-0.0684	0.7865	0.0000	1	-0.0446	0.4599	0.0002	1
hK11	0.0590	0.1247	0.0905	0	-0.0607	0.1200	0.0899	0
GPC1	-0.0312	0.5458	0.0000	1	-0.0140	0.1941	0.0275	0
TFPI 2	-0.0429	0.5290	0.0001	1	-0.0185	0.1092	0.1067	0
hK8	-0.0125	0.0345	0.3847	0	-0.0330	0.1410	0.0644	0
VEGFR 2	-0.0590	0.8644	0.0000	1	-0.0187	0.2535	0.0103	0
LYPD3	0.0434	0.1837	0.0367	0	-0.0294	0.0709	0.1983	0
PODXL	-0.0470	0.7221	0.0000	1	-0.0267	0.5322	0.0001	1
S100A4	-0.0228	0.5661	0.0000	1	-0.0127	0.3086	0.0039	0
IGF1R	-0.0460	0.7877	0.0000	1	-0.0277	0.3060	0.0041	0
ERBB2	-0.0479	0.8570	0.0000	1	-0.0254	0.4982	0.0001	1
ERBB3	-0.0491	0.8696	0.0000	1	-0.0287	0.4949	0.0001	1
SCF	-0.0653	0.7972	0.0000	1	-0.0187	0.0774	0.1781	0
SPARC	-0.0262	0.7222	0.0000	1	-0.0068	0.1231	0.0855	0
GZMH	0.0551	0.1680	0.0467	0	0.0397	0.1352	0.0705	0
TGF alpha	-0.0459	0.4129	0.0007	0	-0.0155	0.0311	0.3994	0
FURIN	-0.0541	0.7849	0.0000	1	-0.0343	0.5472	0.0000	1
CYR61	-0.0618	0.6208	0.0000	1	-0.0345	0.2331	0.0145	0
hK14	0.0162	0.0385	0.3582	0	-0.0551	0.2595	0.0093	0
FADD	0.0179	0.3353	0.0030	0	0.0004	0.0001	0.9613	0
MetAP 2	-0.0159	0.7437	0.0000	1	-0.0080	0.0788	0.1741	0
PVRL4	-0.0392	0.5759	0.0000	1	-0.0302	0.3999	0.0007	0
FASLG	-0.0439	0.4627	0.0003	1	-0.0233	0.1842	0.0323	0
EPHA2	-0.0246	0.6721	0.0000	1	-0.0150	0.3699	0.0013	0

ITGB5	-0.1154	0.8332	0.0000	1	-0.0629	0.5255	0.0000	1
Gal 1	0.0119	0.3020	0.0054	0	-0.0216	0.4916	0.0001	1
SEZ6L	-0.0598	0.7045	0.0000	1	-0.0270	0.3715	0.0012	0
GPNMB	-0.0035	0.0151	0.5676	0	-0.0054	0.0173	0.5312	0
CA9	-0.0082	0.1080	0.1169	0	0.0035	0.0005	0.9261	0
MIA	-0.0299	0.5997	0.0000	1	-0.0154	0.0282	0.4224	0
CTSV	0.0475	0.0872	0.1612	0	-0.0452	0.0979	0.1277	0
CD27	-0.0710	0.8004	0.0000	1	-0.0340	0.3399	0.0022	0
XPNPEP2	-0.0604	0.3677	0.0017	0	-0.0162	0.0238	0.4620	0
ERBB4	-0.0389	0.6769	0.0000	1	-0.0111	0.0365	0.3605	0
HGF	-0.0515	0.5559	0.0000	1	-0.0708	0.4037	0.0006	0
ADAM 8	-0.0742	0.6879	0.0000	1	-0.0522	0.2115	0.0207	0
5 NT	-0.0667	0.5371	0.0000	1	-0.0167	0.0496	0.2848	0
DKN1A	-0.0556	0.5377	0.0000	1	-0.0555	0.3254	0.0029	0
DLL1	-0.0737	0.8428	0.0000	1	-0.0392	0.5567	0.0000	1
MK	-0.0846	0.7743	0.0000	1	-0.0240	0.1418	0.0636	0
ABL1	-0.0836	0.9178	0.0000	1	-0.0349	0.2319	0.0148	0
FGF BP1	0.0247	0.0458	0.3155	0	-0.0129	0.0216	0.4837	0
TLR3	-0.0776	0.7155	0.0000	1	-0.0180	0.0585	0.2440	0
LYN	-0.0181	0.5110	0.0001	1	-0.0004	0.0001	0.9612	0
RET	-0.0289	0.5141	0.0001	1	-0.0179	0.2733	0.0073	0
VIM	-0.0040	0.0378	0.3625	0	0.0182	0.0799	0.1710	0
TNFRSF19	-0.0311	0.4691	0.0002	1	0.0051	0.0014	0.8570	0
CRNN	-0.0593	0.2156	0.0223	0	-0.0363	0.0809	0.1682	0
TCL1A	-0.0037	0.0200	0.5093	0	0.0065	0.1263	0.0813	0
CD160	-0.0382	0.5450	0.0000	1	0.0059	0.0055	0.7256	0
TNFRSF4	-0.0454	0.6999	0.0000	1	-0.0011	0.0000	0.9769	0
MIC AB	-0.0545	0.4061	0.0008	0	-0.0194	0.1824	0.0332	0
WISP 1	-0.0488	0.6470	0.0000	1	-0.0174	0.1868	0.0310	0
CXL17	-0.0643	0.7548	0.0000	1	-0.0355	0.3864	0.0009	0
PPY	-0.0172	0.0826	0.1732	0	0.0133	0.0094	0.6759	0
S100A11	-0.0008	0.0047	0.7510	0	0.0077	0.0384	0.3481	0
AR	-0.0178	0.1592	0.0534	0	-0.0252	0.2386	0.0132	0
ESM 1	-0.0427	0.7518	0.0000	1	-0.0273	0.2999	0.0046	0
CD207	-0.0199	0.6107	0.0000	1	-0.0041	0.0163	0.6134	0
ICOSLG	-0.0005	0.0006	0.9105	0	-0.0160	0.2862	0.0059	0
WFDC2	-0.0502	0.4818	0.0002	1	-0.0274	0.1643	0.0444	0
CXCL13	-0.0514	0.5966	0.0000	1	-0.0197	0.1503	0.0555	0
MAD homolog 5	-0.0491	0.6786	0.0000	1	-0.0095	0.0828	0.1630	0
ADAM TS 15	-0.0495	0.6927	0.0000	1	-0.0221	0.4463	0.0003	1
CD70	-0.0148	0.2815	0.0077	0	-0.0079	0.0812	0.5356	0
RSPO3	-0.0230	0.4720	0.0002	1	-0.0064	0.0342	0.3763	0
FR gamma	-0.0855	0.0878	0.1598	0	0.0097	0.0026	0.8075	0
CEACAM5	0.0286	0.1295	0.0841	0	-0.0306	0.0836	0.2959	0
VEGFR 3	-0.0329	0.4558	0.0003	1	-0.0267	0.3200	0.0032	0
MUC 16	-0.0500	0.4479	0.0003	1	-0.0153	0.0410	0.3318	0

WIF 1	-0.0697	0.8562	0.0000	1	-0.0368	0.5401	0.0000	1
GZMB	0.0230	0.1115	0.1107	0	0.0214	0.1418	0.0636	0
FCRLB	-0.0064	0.2469	0.0135	0	0.0000	0.0000	0.9970	0
ANXA1	-0.0027	0.0539	0.2749	0	-0.0092	0.2235	0.0170	0
FR alpha	-0.0363	0.5176	0.0001	1	-0.0525	0.4966	0.0001	1

Supplemental Table S6. Parameter values from ANOVA tests of linear regression models calculated individually from NPX values (log_2) for all 92 proteins measured for ULSAM plasma and serum samples stored at -70°C. **β** describes the slope of the linear model in the unit NPX per year. **VarExp** describes the estimated proportion of variation explained by the model. **P-val** represents the probability of the model. **Signif** is set to 1 if the parameter p-val is below the limit for testing of multiple hypotheses (0.05/92) and β is negative. Fresh control samples were used to evaluate effects of age of individuals and sample type (plasma/serum) on protein abundance. **P-val Age** describes the probability that there was a correlation between measured protein abundance and age of individuals. **P-val Type** describes the probability that there was a difference between measured protein abundance in plasma and serum. **Signif Age** and **Signif Type** is set to 1 if the parameters p-val Age and p-val Type, respectively is nominally significant (<0.05). These nominally significant models for individual age and sample type dependence evaluate from the control samples were used to normalize the ULSAM samples.

Protein	β	VarExp	p-val	Signif	p-val Age	Signif Age	p-val Type	Signif Type
TXLNA	-0.0233	0.0693	0.0155	0	0.0183	1	0.6051	0
VEGF A	-0.0165	0.1235	0.0010	0	0.6848	0	0.0000	1
CPE	0.0199	0.2238	0.0000	0	0.0120	1	0.1451	0
KLK13	0.0091	0.0336	0.0952	0	0.0081	1	0.3543	0
CEACAM1	0.0009	0.0059	0.4883	0	0.3024	0	0.0000	1
MSLN	0.0113	0.0331	0.0978	0	0.0011	1	0.3842	0
TNFSF13	-0.0065	0.0504	0.0400	0	0.0000	1	0.1896	0
EGF	0.0164	0.0319	0.1041	0	0.9897	0	0.0000	1
TNFRSF6B	-0.0016	0.0009	0.7810	0	0.0001	1	0.0004	1
SYND1	-0.0004	0.0001	0.9265	0	0.0956	0	0.0033	1
TGFR 2	-0.0013	0.0014	0.7334	0	0.0000	1	0.0036	1
IL 6	-0.0071	0.0060	0.4852	0	0.0562	0	0.1708	0
CD48	0.0021	0.0046	0.5418	0	0.0003	1	0.0081	1
SCAMP3	0.0050	0.0020	0.6866	0	0.0885	0	0.2925	0
LY9	0.0104	0.0750	0.0117	0	0.0003	1	0.1385	0
IFN gamma R1	0.0010	0.0015	0.7234	0	0.0000	1	0.0020	1
ITGAV	0.0055	0.0526	0.0359	0	0.0816	0	0.5866	0
TNFSF10	-0.0056	0.0191	0.2099	0	0.0178	1	0.0062	1
hK11	0.0024	0.0046	0.5418	0	0.0000	1	0.0373	1
GPC1	0.0053	0.0310	0.1092	0	0.0000	1	0.0019	1
TFPI 2	0.0025	0.0047	0.5343	0	0.0000	1	0.3731	0
hK8	0.0043	0.0201	0.1982	0	0.0067	1	0.0127	1
VEGFR 2	0.0035	0.0397	0.0693	0	0.0035	1	0.0075	1

LYPD3	0.0045	0.0197	0.2030	0	0.4761	0	0.0587	0
PODXL	-0.0022	0.0176	0.2291	0	0.4119	0	0.0003	1
S100A4	-0.0167	0.2114	0.0000	1	0.5728	0	0.0000	1
IGF1R	0.0020	0.0075	0.4344	0	0.0000	1	0.0000	1
ERBB2	0.0011	0.0016	0.7172	0	0.0912	0	0.0042	1
ERBB3	0.0039	0.0474	0.0466	0	0.0656	0	0.0000	1
SCF	-0.0072	0.0242	0.1574	0	0.0302	1	0.0025	1
SPARC	-0.0029	0.0393	0.0706	0	0.3035	0	0.0000	1
GZMH	-0.0060	0.0089	0.3933	0	0.0003	1	0.0008	1
TGF alpha	-0.0334	0.2866	0.0000	1	0.9043	0	0.0000	1
FURIN	-0.0055	0.0354	0.0864	0	0.5024	0	0.0000	1
CYR61	0.0100	0.0490	0.0431	0	0.0000	1	0.0003	1
hK14	0.0106	0.0587	0.0264	0	0.0005	1	0.2873	0
FADD	-0.0051	0.0065	0.4657	0	0.4697	0	0.0079	1
MetAP 2	-0.0133	0.0624	0.0219	0	0.0884	0	0.0341	1
PVRL4	0.0029	0.0049	0.5274	0	0.0000	1	0.0464	1
FASLG	0.0003	0.0000	0.9521	0	0.8525	0	0.8766	0
EPHA2	0.0022	0.0051	0.5183	0	0.0000	1	0.0408	1
ITGB5	0.0060	0.0450	0.0528	0	0.0001	1	0.0018	1
Gal 1	-0.0041	0.0548	0.0320	0	0.0020	1	0.0036	1
SEZ6L	0.0008	0.0012	0.7533	0	0.0000	1	0.0001	1
GPNMB	0.0021	0.0207	0.1914	0	0.0001	1	0.0066	1
CA9	0.0032	0.0025	0.6496	0	0.0064	1	0.1305	0
MIA	-0.0008	0.0017	0.7058	0	0.0002	1	0.0059	1
CTSV	0.0075	0.0326	0.1005	0	0.0017	1	0.0461	1
CD27	0.0007	0.0003	0.8836	0	0.0000	1	0.0766	0
XPNPEP2	0.0119	0.0330	0.0979	0	0.0550	0	0.2121	0
ERBB4	0.0040	0.0213	0.1856	0	0.0941	0	0.0006	1
HGF	-0.0186	0.2024	0.0000	1	0.0242	1	0.0000	1
ADAM 8	-0.0114	0.1413	0.0004	1	0.0097	1	0.0000	1
5 NT	-0.0113	0.0425	0.0600	0	0.0598	0	0.0001	1
DKN1A	-0.0025	0.0007	0.8109	0	0.1772	0	0.8108	0
DLL1	-0.0001	0.0000	0.9612	0	0.0000	1	0.0044	1
MK	-0.0066	0.0149	0.2685	0	0.0000	1	0.5972	0
ABL1	-0.0055	0.0099	0.3668	0	0.0029	1	0.3502	0
FGF BP1	0.0125	0.1253	0.0010	0	0.0253	1	0.1022	0
TLR3	0.0162	0.0727	0.0131	0	0.6730	0	0.1478	0
LYN	-0.0006	0.0001	0.9274	0	0.1001	0	0.0351	1
RET	-0.0018	0.0031	0.6146	0	0.0002	1	0.0000	1
VIM	-0.0182	0.0991	0.0035	0	0.0011	1	0.0000	1
TNFRSF19	-0.0015	0.0010	0.7759	0	0.0000	1	0.0066	1
CRNN	0.0003	0.0000	0.9682	0	0.5959	0	0.2916	0
TCL1A	-0.0183	0.0276	0.1308	0	0.0793	0	0.0004	1
CD160	0.0013	0.0006	0.8316	0	0.0000	1	0.1371	0
TNFRSF4	-0.0022	0.0026	0.6481	0	0.0000	1	0.0394	1
MIC AB	-0.0046	0.0014	0.7310	0	0.3235	0	0.5504	0

WISP 1	-0.0005	0.0001	0.9270	0	0.0000	1	0.0000	1
CXL17	0.0054	0.0125	0.3114	0	0.0000	1	0.0002	1
PPY	-0.0394	0.1051	0.0026	0	0.0054	1	0.8085	0
S100A11	-0.0161	0.1072	0.0024	0	0.0569	0	0.2343	0
AR	-0.0010	0.0009	0.7830	0	0.0000	1	0.0092	1
ESM 1	-0.0007	0.0004	0.8564	0	0.0001	1	0.3438	0
CD207	0.0040	0.0126	0.3088	0	0.0000	1	0.0140	1
ICOSLG	0.0041	0.0271	0.1344	0	0.0011	1	0.0000	1
WFDC2	0.0006	0.0002	0.8869	0	0.0000	1	0.0294	1
CXCL13	-0.0098	0.0357	0.0851	0	0.0005	1	0.0085	1
MAD homolog 5	0.0021	0.0095	0.3788	0	0.0179	1	0.1693	0
ADAM TS 15	-0.0017	0.0021	0.6757	0	0.0017	1	0.0000	1
CD70	-0.0050	0.0132	0.2980	0	0.0031	1	0.0087	1
RSPO3	-0.0203	0.1319	0.0007	0	0.0000	1	0.4910	0
FR gamma	-0.0575	0.0918	0.0051	0	0.3064	0	0.5196	0
CEACAM5	0.0058	0.0055	0.5038	0	0.0099	1	0.4080	0
VEGFR 3	-0.0029	0.0310	0.1090	0	0.1731	0	0.0000	1
MUC 16	0.0022	0.0008	0.7983	0	0.2216	0	0.1328	0
WIF 1	0.0013	0.0017	0.7102	0	0.0050	1	0.0000	1
GZMB	-0.0070	0.0176	0.2295	0	0.1547	0	0.0012	1
FCRLB	-0.0138	0.0549	0.0319	0	0.9737	0	0.6774	0
ANXA1	-0.0324	0.2579	0.0000	1	0.3169	0	0.4410	0
FR alpha	-0.0015	0.0013	0.7409	0	0.0000	1	0.0195	1
